# Incidence, causes, and consequences of preventable adverse drug reactions occurring in inpatients: A systematic review of systematic reviews

**DOI:** 10.1371/journal.pone.0205426

**Published:** 2018-10-11

**Authors:** Dianna Wolfe, Fatemeh Yazdi, Salmaan Kanji, Lisa Burry, Andrew Beck, Claire Butler, Leila Esmaeilisaraji, Candyce Hamel, Mona Hersi, Becky Skidmore, David Moher, Brian Hutton

**Affiliations:** 1 Clinical Epidemiology Program, Ottawa Hospital Research Institute, Ottawa, Ontario, Canada; 2 Department of Pharmacy, The Ottawa Hospital, Ottawa, Ontario, Canada; 3 Leslie Dan Faculty of Pharmacy, University of Toronto, Toronto, Ontario, Canada; 4 School of Epidemiology, Public Health and Preventive Medicine, University of Ottawa, Ottawa, Ontario, Canada; Newcastle University, UNITED KINGDOM

## Abstract

**Background:**

Preventable adverse drug reactions (PADRs) in inpatients are associated with harm, including increased length of stay and potential loss of life, and result in elevated costs of care. We conducted an overview of reviews (i.e., a systematic review of systematic reviews) to determine the incidence of PADRs experienced by inpatients. Secondary review objectives were related to assessment of the effects of patient age, setting, and clinical specialty on PADR incidence.

**Methods:**

The protocol was registered in PROSPERO (CRD42016043220). We performed a search of Medline, Embase, and the Cochrane Library, limiting languages of publication to English and French. We included published systematic reviews that reported quantitative data on the incidence of PADRs in patients receiving acute or ambulatory care in a hospital setting. The full texts of all primary studies for which PADR data were reported in the included reviews were obtained and data relevant to review objectives were extracted. Quality of the included reviews was assessed using the AMSTAR-2 tool. Both narrative summaries of findings and meta-analyses of primary study data were undertaken.

**Results:**

Thirteen systematic reviews encompassing 37 unique primary studies were included. Across primary studies, the PADR incidence was highly varied, ranging from 0.006 to 13.3 PADRs per 100 patients, with a pooled incidence estimate of 0.59 PADRs per 100 patients. Substantial heterogeneity was present across both reviews and primary studies with respect to review/study objectives, patient age, hospital setting, medical discipline, definitions and assessment tools used, event detection methods, endpoints of interest, and units of measure. Thirteen primary studies used prospective event detection methods and had a pooled PADR incidence of 3.13 (2.87–3.38) PADRs per 100 patients; however, extreme statistical heterogeneity (I^2^ = 97%) indicated this finding should be considered with caution. Subgroup meta-analyses demonstrated that PADR incidence varied significantly with event detection method (prospective > retrospective > voluntary reporting methods), hospital setting (ICU > wards), and medical discipline (medical > surgical). High statistical heterogeneity (I^2^ > 80%) was present across all analyses, indicating results should be interpreted with caution. Effects of patient age could not be assessed due to poor reporting of age groups used in primary studies.

**Discussion:**

The method of event detection appeared to significantly influence PADR incidence, with prospective methods having the highest reported PADR rate. This finding is in agreement with the background literature. High methodological and statistical heterogeneity across primary studies evaluating adverse drug events reduces the validity of the overall PADR incidence derived from the meta-analyses of the pooled data. Data pooled from studies using only prospective methods of event detection should provide an overall estimate closest to the true PADR incidence; however, our estimate should be considered with caution due to the statistical heterogeneity found in this group of studies. Future studies should employ prospective methods of detection. This review demonstrates that the true overall incidence of PADRs is likely much greater than the overall pooled incidence estimate of 0.59 PADRs per 100 patients obtained when event detection method was not taken into consideration.

## Introduction

**Fig 1:**
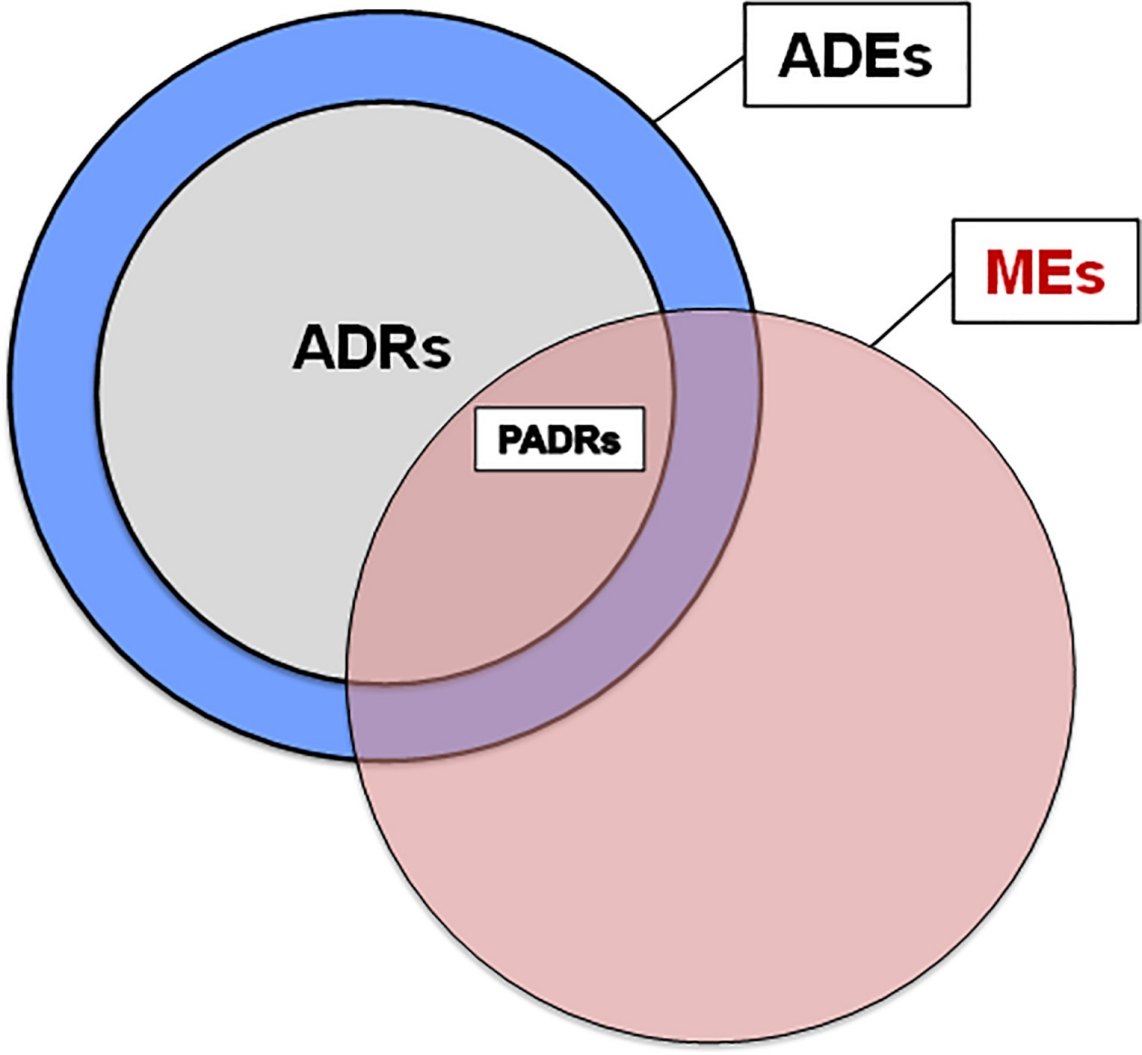
Venn diagram of adverse event definitions.

Medical errors are the third leading cause of death in the United States [1]. In the year 2000, an estimated 70,000 Canadian patients experienced at least one highly preventable adverse event (AE) due to health care management, resulting in an estimated 9,250 to 23,750 preventable deaths [2]. Medication errors (MEs)—failures in the treatment process that lead to, or have the potential to lead to, harm to the patient [3]—accounted for almost a quarter of all AEs [2]. Adverse events caused by medication errors are generally considered to be preventable [4], and are referred to as *preventable adverse drug events* (PADEs). Adverse drug events (ADEs), by definition [5], occur after administration of a medication at any dosage level and may or may not incur harm to the patient (e.g., over-dosage of a drug that caused increased monitoring of a patient but no resultant harm). Adverse drug reactions (ADRs) are, by definition [6], a subset of ADEs (Fig 1**)** in that they only occur following drug administration within normal dose ranges and they result in “noxious and unintended” consequences to the patient. Preventable adverse drug reactions (PADRs) include ADRs caused by medication errors, whether they be acts of omission or commission, incorrect medication/dose/timing, administration of a medication to a patient with a known allergy, inadequate monitoring, or other errors.

The assumption of preventability suggests that appropriate healthcare interventions directed at the root causes of MEs could reduce the incidence of patient harm. PADRs occurring in healthcare facilities such as acute care hospitals may be easier to address through interventions than community-acquired PADRs because the setting is more controlled. Enhanced knowledge of why, how, when, and where PADEs/PADRs occur in hospitals could inform the development of these interventions, ultimately improving patient care and reducing healthcare system burdens.

Pharmacovigilance in Canada relies on a spontaneous (i.e., voluntary) ADR reporting system [7]. Spontaneous reporting systems are limited by under-reporting, with potentially less than 10% of ADRs being accounted for [7,8]. Poor quality of the ADR reports submitted also reduces the utility of spontaneous systems, as essential information to evaluate the likelihood of an event being caused by a drug may be lacking [7]. Furthermore, the incidence of reactions due to individual drugs cannot be calculated because the number of patients taking a drug in the population is unavailable, and report duplication is common [7].

To address some current gaps in pharmacovigilance in Canada, “Vanessa’s Law” was passed in 2014, though the law is not in full effect because changes to the *Food and Drug Act* are still pending [9]. One of the mandates of Vanessa’s Law is to make reporting of serious ADRs mandatory by healthcare facilities in Canada (currently, only drug manufacturers are required to submit ADR reports). However, even under a mandatory reporting system, the rate of ADR submissions may be inadequate to effectively detect drug safety signals, as evidenced by reviews of pharmacovigilance in New Zealand and France [10]. Methods to acquire information on ADRs and PADRs are needed outside of the federal regulatory and reporting system, if we wish to have a better understanding of PADR occurrence in specific settings and populations.

We undertook an overview of reviews (i.e., a systematic review of systematic reviews) to determine the incidence of inpatient PADRs worldwide and in Canada specifically. Secondary review objectives included assessment of the effects of patient age, setting (e.g., intensive care units, wards), and clinical specialty (e.g., medical, surgical) on inpatient PADR incidence, as well as a description of various stages of the medication process at which errors occurred that led to PADRs, system-level causes (e.g., lack of quality control, lack of staff education), and levels of severity of the observed PADRs.

## Methods

Preliminary scoping of the literature identified several existing systematic reviews on the topic of ADRs. Therefore, we used an “overview of reviews” approach to identify relevant data [11,12]. Methods were developed in consultation with both the Cochrane Handbook’s chapter on overviews of reviews [11] and recent work by Smith [12]. A review protocol [13] was drafted prior to review initiation and registered with PROSPERO (CRD42016043220). Amendments were made to this protocol after completion of the overview of reviews, when we recognized that limitations in reporting of PADR data within the included systematic reviews had precluded answering of many of our secondary review questions. In this manuscript, the term “reviews” refers to the systematic reviews included under the original protocol, while “studies” refers to the primary studies found within those reviews.

### Research questions addressed

This review was designed to answer the following primary research question: What is the incidence of PADRs in acute and continuing/long-term care hospitals/institutions (including both academic and community hospitals)? A series of secondary review objectives were also addressed: (1) assessment of PADR incidence within different patient age groups (e.g., pediatric, adult, and elderly patients), settings (e.g., acute, continuing, and long-term care; academic vs community hospitals; wards vs ICUs), and clinical specialities (e.g., medicine vs surgery); (2) causes of PADRs: descriptions of the stages of the medication process at which errors occurred and system-level causes of PADRs (e.g., lack of quality control, lack of staff education); (3) a description of the severity of patient outcomes associated with PADRs; and (4) a description of what drugs or drug classes are commonly reported to be associated with PADRs.

### Study eligibility criteria

Eligibility criteria to identify relevant systematic reviews for this review were underpinned by the population-intervention-comparator-outcomes-study design (PICOS) framework. Criteria were as follows:

- **Population**: Reviews of primary studies involving patients receiving acute or ambulatory care from hospitals and being treated with drug therapy were included. Reviews with studies set in other institutional settings such as long-term care facilities were also included; however, primary care settings were not eligible. Inpatient data reported in comparisons of inpatient vs outpatient settings or hospital-based vs other settings were also eligible.
- **Intervention/comparators**: No specific interventions or comparators were necessary for inclusion.
- **Outcomes**: A specific definition of PADR or PADE was not established as an a priori eligibility criterion. We anticipated definitions to vary by review and study, and that these variations would likely be highly associated with results. Assessment of an association was part of our data synthesis. Similarly, a formal assessment of causality between a drug and an event was not necessary for eligibility. Reviews reporting on the occurrence of adverse reactions following adequate administration of medication were not eligible, unless the events were deemed preventable (e.g., a patient with a previously recognized drug reaction). Reviews reporting data on ameliorable ADRs (i.e., those that could not have been prevented but whose severity could have been reduced) or PADRs related to herbal or non-prescription medications were included, if the events occurred in an inpatient setting. Although PADR incidence was the primary outcome of interest, all PADR measures were eligible, with secondary outcomes including the distribution of causes of PADRs (i.e., stages of the medication process at which errors occurred, lack of staff education, lack of quality control mechanisms, etc.), and evaluations of PADR severity.
- **Study design**: Only systematic review designs were included. We defined a systematic review as being a review with a clearly specified review question, that incorporates a systematic search of one or more electronic literature databases, clearly defined eligibility criteria, systematic study selection and data collection by two or more reviewers, an appraisal of the risk of bias of included studies, and a synthesis of all information using a quantitative or qualitative approach. Reviews not meeting these criteria were excluded.

### Searching the literature for relevant reviews

A search strategy was developed in collaboration with an experienced information specialist (BS) and independently peer-reviewed by a second information specialist using PRESS criteria [14]. We searched Medline, Embase, and the Cochrane Library on 2 June 2016, without date restrictions, but limiting languages to English and French. Key search terms included *adverse drug reaction reporting systems*, *drug-related side effects and adverse reactions*, *medication errors*, and an in-depth list of synonyms, given the variable terminology in the area. The full search strategies have been provided in S1 Text.

### Process of study selection

The titles and abstracts of all citations identified in the literature searches were screened independently by two reviewers, using pre-defined criteria. The full texts of citations identified as potentially relevant were then screened using a similar process, with disagreements settled through consultation with a third review team member. Screening forms were developed in online systematic review software (DistillerSR, Evidence Partners Inc., Ottawa, Canada), and piloting of the forms was performed to ensure similar understanding of the eligibility criteria amongst reviewers.

To ensure as broad a scope of content as possible, we included all systematic reviews, including updates of previous systematic reviews and systematic reviews with overlapping evidence bases (i.e., primary studies could appear within more than one included systematic review).

### Data extraction and risk-of-bias assessment

A data collection form was developed in Microsoft Excel software (Microsoft Corporation, Seattle, USA) and piloted on a small number of studies. Following refinement of the data extraction form, two review team members conducted data extraction independently, with a third member consulted when disagreements occurred. The data extracted were comprehensive in scope as we were addressing multiple review objectives; a table summarizing the details of all information gathered is provided in S2 Text. Methodological quality/risk of bias of the included systematic reviews was assessed using the AMSTAR 2 tool [15].

### Synthesis of data

A descriptive approach was used to synthesize the data extracted from the included reviews. Fundamental features of the reviews and the review findings were summarized narratively, supplemented with graphics to facilitate interpretation and to highlight key evidence. A citation network diagram of the included reviews and their primary studies was developed to illustrate the connectedness of the available literature, highlighting the degree of overlap in the evidence base across reviews. Raw PADR data reported in reviews were converted to PADR incidence rates, with common units of measure, including (1) PADRs per 100 patients, (2) PADRs per 1,000 patient-days, (3) percentage of patients experiencing at least one PADR, (4) PADRs per 1,000 doses, and (5) “other” units. PADR incidence rates reported in other units were converted to these common units (e.g., PADRs per resident-months were converted to PADRs per 1,000 patient-days; PADRs per 100 admissions or per 100 discharges were assumed to be equivalent to PADRs per 100 patients). The Jadad framework for discordant reviews [16] was applied to assess potential causes of variations in PADR incidences reported in the included reviews; an overview of its contents are provided in S3 Text.

### Protocol amendments: Retrieval and synthesis of primary study data

Upon finding that there were challenges in answering many of our review questions using only data that were reported in systematic reviews, we amended our protocol to incorporate methods for gathering additional information. We obtained the primary studies reported as having PADR data in the included systematic reviews and screened them for relevance using the same criteria that were employed for the reviews. Primary studies that did not report PADR incidence data were excluded. Following relevance screening, two reviewers independently extracted relevant data directly from the primary studies. Descriptive characteristics of the included primary studies were summarized narratively and graphically, where appropriate. Raw PADR data and PADR incidence rates reported in the primary studies were converted to common units as described earlier. Meta-analyses were conducted to calculate pooled PADR incidence rates for the units of measure that had been reported in sufficient numbers of studies to allow synthesis. Random-effects models were fit to account for high heterogeneity between studies (^2^) in the estimation of mean PADR incidence [17].

The pooled PADR incidence rate across studies was considered an inaccurate estimate of the true rate, given the influence of event detection method [18–20] and the numerous methods of event detection found in our included studies. A subgroup meta-analysis was conducted to provide a more accurate estimate of PADR incidence and to describe the influence of event detection method categories on PADR incidence (i.e., voluntary reporting, retrospective chart review, prospective methods, and other methods). Studies included in the “voluntary reporting” category used strictly voluntary or stimulated voluntary reporting methods (i.e., promotion of voluntary reporting by various methods, including researchers asking about new cases, meetings with medical personnel to stress the importance of reporting and/or the study, research conducted jointly with patient record review departments, or pharmacist participation in the form of organized lectures, group discussions, etc. [18,19]). “Retrospective chart review” occurred after patient discharge and may or may not have been aided by manual or computerized trigger tools (e.g., a validated ADE trigger tool designed by the Institute for Healthcare Improvement (IHI) [21]) or may have been retrospective evaluations of computer-generated alerts based on pharmacy and laboratory signals [22]. “Prospective methods” must have been implemented before patient discharge and could include chart review and/or patient/healthcare team interviews, preferably on a daily basis [18–20]. “Other” event detection methods did not fit into the preceding categories. Categories with non-overlapping 95% confidence intervals were considered to have significantly different PADR incidence rates. Subgroup heterogeneity was evaluated using the I^2^ statistic [23]. The “prospective methods” category was considered by the research team to most closely estimate the true incidence of PADRs in the population as it is known to be the most sensitive event detection method [18–20].

To address secondary review questions, we conducted additional subgroup meta-analyses to identify differences in PADR incidence between categories of patient age (i.e., paediatric, adult, geriatric, all ages, and not reported), setting (i.e., wards and ICUs), and medical discipline (i.e., medicine and surgery). Categories were not established a priori and instead were based upon the reporting structure and availability of data in the primary studies. Studies self-identifying as “paediatric,” “adult,” or “geriatric” studies were categorized as such; we did not impose age limits on the categories. The “adult” age category included at least some geriatric patients, with patients >65 years making up the majority of “adult” patients in some studies. We could not further refine the “adult” age category based on the patient age data reported. Significant differences between subgroups were identified by non-overlapping confidence intervals. Graphical interpretations of the data have been presented, where appropriate.

All other secondary review questions were addressed through narrative synthesis and tabular presentation of the data. To assess system-level causes of PADRs, we looked at intervention studies and categorized them into groups according to the system-level intervention they evaluated: computerized physician order entry (CPOE), clinical decision support systems (CDSS), pharmacist participation, automation of drug dispensing or administration, and institutional cultural interventions, which included staff education, etc. Because we did not conduct a systematic review of primary studies evaluating the effects of these interventions, we recognized that our list of included studies was not comprehensive: our small sample of intervention studies may not reflect the overall literature base and, therefore, meta-analyses may be biased. Thus, we did not conduct meta-analyses, and instead the effect on PADR incidence of each intervention group was crudely assessed by vote-counting—i.e., comparing the number of studies in each intervention group that demonstrated a significant decrease in PADR incidence post-intervention, no significant change, or a decreased incidence but the significance was not reported.

All meta-analyses and subgroup meta-analyses were fit using random-effects models, using Comprehensive Meta-analysis software (Version 3.3.070; Biostat, Inc., Englewood, NJ, USA).

### Reporting of review findings

Drafting of this manuscript was guided by the PRISMA Statement [24] and a PRISMA Checklist is available, documenting the completeness of reporting (see S4 Text).

## Results

Evidence identified from the review is presented in two stages. The findings from the overview of reviews have been presented first, followed by the more substantive evidence obtained from the primary studies that were reported in our included reviews.

### Review characteristics

**Fig 2:**
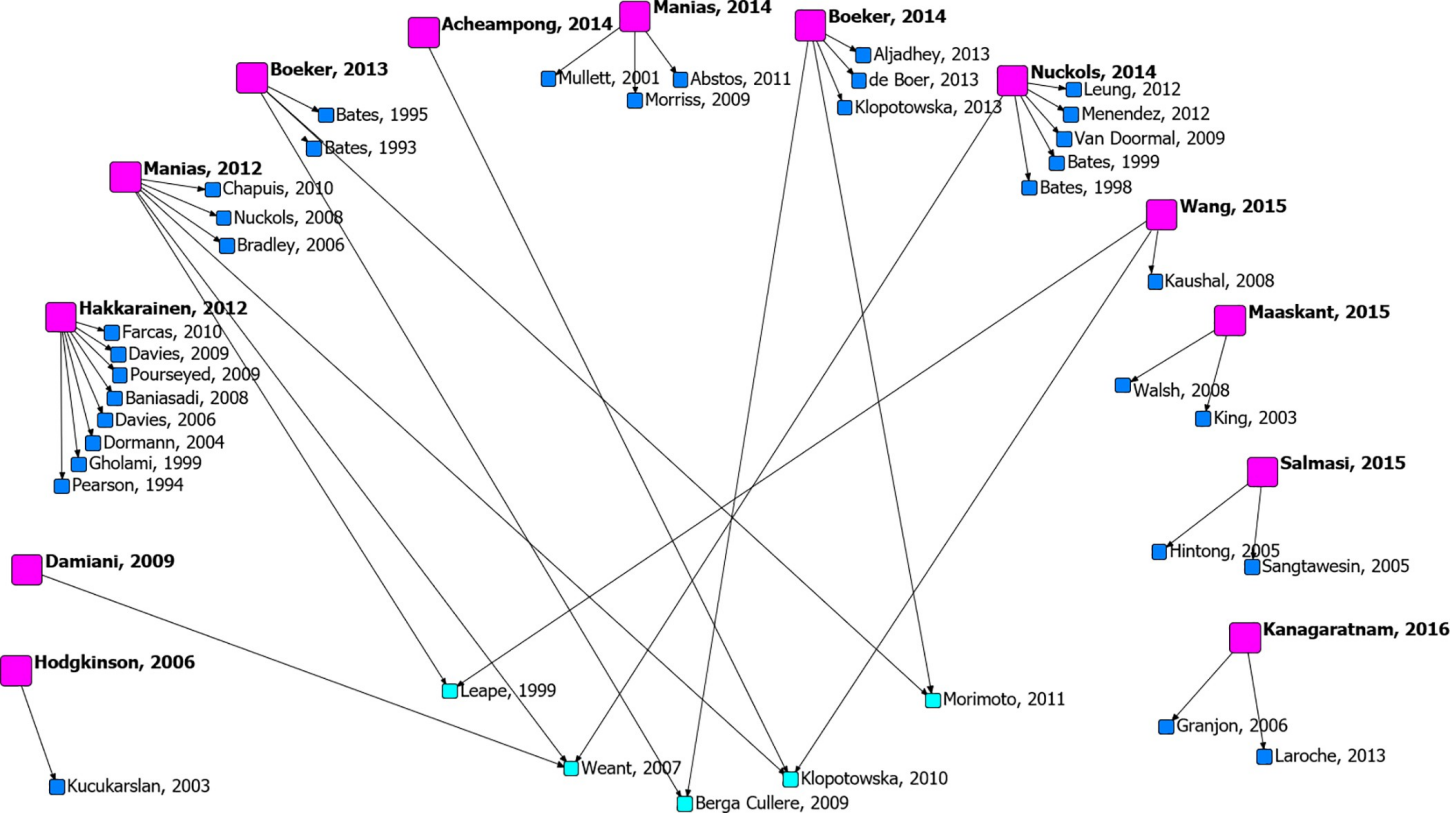
Citation network diagram of included systematic reviews with included primary studies that reported PADR data. Pink = systematic review; dark blue = primary study reported in only one systematic review; light blue = primary study reported in two or more systematic reviews.

A flow diagram summarizes the process of review selection and identification of the primary studies within the reviews (see S1 Fig). Thirteen reviews were included [25–37] that reported PADR data from 37 primary studies [38–74]. A citation map of the evidence demonstrates there was limited commonality of primary studies amongst the included reviews (Fig 2); only five of 37 primary studies (14%) were common to two or more reviews [70–74].

Characteristics of the 13 included systematic reviews and the 37 primary studies that they included are summarized in Tables 1 and 2, respectively. Detailed characteristics of the systematic reviews are reported in Table 3. Eight reviews aimed to assess interventions to reduce MEs, ADRs, or PADRs [26,28–31,33–35], while five evaluated incidence, prevalence, or risk factors for MEs, ADRs, or PADRs as their stated objective [25,27,32,36,37]. Review eligibility criteria related to patient age, hospital setting, and unit of measure were highly varied.

**Table 1:** Summary characteristics of included systematic reviews and primary studies.

Characteristic and Categories		Reviews (n = 13)	Primary Studies (n = 37)
**Year of publication**	1990–1999	0 (0%)	7 (19%)
	2000–2009	2 (15%)	21 (57%)
	2010–2017	11 (85%)	9 (24%)
**Study design**	Interventional	8 (62%)	20 (54%)
	Other design^a^	5 (38%)	17 (46%)
**Patient age**	Paediatric only	2 (15%)	6 (16%)
	Adult	5 (38%)	15 (41%)
	Geriatric only	2 (15%)	4 (11%)
	All ages	4 (31%)	2 (5%)
	Unclear	0 (0%)	10 (27%)
**Hospital setting**	All settings (ICUs and wards)	6 (46%)	13 (35%)
	ICU only	2 (15%)	9 (24%)
	Wards only	0 (0%)	10 (27%)
	PICU	1 (8%)	0 (0%)
	Surgical inpatient settings	1 (8%)	0 (0%)
	All settings except emergency department	1 (8%)	0 (0%)
	“Acute, subacute, and residential care”	1 (8%)	0 (0%)
	Geriatric hospital and non-hospital settings	1 (8%)	0 (0%)
	Long-term care	0 (0%)	3 (8%)
	Anaesthesia only	0 (0%)	1 (3%)
	Not reported	0 (0%)	1 (3%)
**Medical discipline**	Medicine only	NA	11 (30%)
	Surgery only	NA	4 (11%)
	Medicine and surgery	NA	21 (57%)
	Unclear	NA	1 (3%)
**Endpoint of interest (reviews) or event detected (primary studies)**	Medication errors	8 (62%)	11 (30%)
	ADRs/ADEs	2 (15%)	17 (46%)
	PADRs/PADEs	3 (23%)	1 (3%)
	Medication errors and ADRs/ADEs	0 (0%)	8 (22%)
**Event detection method**	Voluntary/stimulated voluntary	NA	9 (24%)
	Retrospective chart review/alert evaluation	NA	10 (27%)
	Prospective methods	NA	15 (41%)
	Other	NA	3 (8%)
**PADR incidence unit of measure reported**	PADRs/100 patients	7 (54%)	32 (86%)
	PADRs/1,000 patient-days	7 (54%)	16 (43%)
	Percentage of patients experiencing at least one PADR	4 (31%)	3 (8%)
	PADRs/1,000 doses	4 (31%)	6 (16%)
	Other units^b^	2 (15%)	3 (8%)

^a^ Other designs included estimates of incidence or prevalence and risk factor analyses

^b^ Other units included the number of PADRs/100 detailed opportunities for error and the number of PADRs/1,000 medication orders

**Table 2:** Additional characteristics of included primary studies.

Characteristic and Categories		Primary Studies (n = 37)
**ADR/ADE definition used**	“Injury from a drug”	12 (32%)
	WHO [1]	8 (22%)
	Edwards and Aronson [2]	2 (5%)
	NCC MERP [3] categories E–H	4 (11%)
	NCC MERP [3] categories E–I	4 (11%)
	NCC MERP [3] categories F–I	1 (3%)
	NCC MERP [3] categories D–I	1 (3%)
	Custom definition	2 (5%)
	Not reported	3 (8%)
**Causality assessment tool used**	Naranjo [4]	8 (22%)
	Brigham and Women’s Hospital [5]	3 (8%)
	French Causality Assessment Tool [6]	1 (3%)
	Modified Karch and Lasagna tool [7]	1 (3%)
	WHO-Uppsala Monitoring Centre Tool [2,8]	1 (3%)
	Custom tool	3 (8%)
	Not used/reported	20 (54%)
**Preventability assessment tool used**	All medication errors are preventable	14 (38%)
	Schumock and Thornton (and adaptations) [9]	6 (16%)
	Dubois and Brook [10]	5 (14%)
	Hallas [11]	2 (5%)
	French Adverse Drug Reactions Preventability Scale [12]	1 (3%)
	Custom tool	4 (11%)
	Not used/reported	6 (16%)
**Sample size enrolled**	0–249 patients	5 (14%)
	250–499 patients	9 (24%)
	500–999 patients	4 (11%)
	1,000–9,999 patients	11 (30%)
	10,000–20,000 patients	1 (3%)
	30,000–35,000 patients	3 (8%)
	>200,000 patients	1 (3%)
	Not reported	3 (8%)

**Table 3:** Detailed characteristics of the 13 included systematic reviews (focused on elements of the Jadad framework).

Review first author/year; funding source^a^	Number of included studies (number reporting PADR incidence)	Objective	Patient age of interest	Hospital setting of interest	Study designs of interest	Other selection criteria of the primary studies	Search strategy: number of databases; date range; language restriction^b^	PADR incidence unit of measure^c^	Other review errors or differences potentially affecting reported PADR incidence
Kanagaratnam 2016 [13]; α	6 (2)	To describe ADR prevalence in geriatrics with cognitive disorders	Geriatric	Hospital and non-hospital settings	Intervention or observational	Elderly patients with cognitive disorders or dementia syndrome	5; To 4 Feb 2015; π	χ	One study included “probable or likely” PADRs and included only PADRs present at admission (not in-hospital PADRs) [14]. One study may have included PADRs occurring prior to admission [15].
Boeker 2015 [16]; α	4 (5)	To identify patient characteristics and medication types associated with ADE/PADEs during admission	Adult	All hospital settings except emergency	Any	Studies for which individual patient data were available	2; 2000–2011; π	ϕ, χ	One study included PADRs present at admission—it is unclear if the reviewers excluded these IPD [17].
Maaskant 2015 [18]; β	7 (2)	To assess effectiveness of interventions to reduce MEs and related harms in hospitalized children	Pediatric	All hospital settings	RCTs, non-RCTs, controlled longitudinal, interrupted time-series evaluating CPOEs	None	14; 1947–2014; μ	λ	Unclear if the incidence reported in one study was of PADRs or of all ADRs [19].
Salmasi 2015 [20]; β	17 (2)	To estimate prevalence of MEs in Southeast Asian countries	No age restriction	Southeast Asian only; otherwise not reported	RCTs, non-RCTs, longitudinal, cohort, case-control, or descriptive	None	Not reported; To 2014; π	ϕ	One study included only anaesthesia patients [21].
Wang 2015 [22]; θ	13 (3)	To assess effect of ICU pharmacist interventions on MEs	Not reported	ICU only	Non-RCTs (controlled longitudinal, historical control, cohort) evaluating a pharmacist intervention	Excluded if effect of intervention on ME and PADEs not clearly reported	3; To Aug 2014; τ	λ	Review did not include control unit data in baseline PADR incidence for 2 studies [23,24]. One study included MEs that did not cause harm in their definition of PADRs, inflating PADR incidence [23].
Acheampong 2014 [25]; θ	42 (1)	To review literature of interventions for medication safety in hospitals	Not reported	All hospital settings	Intervention	None	8; To April 2013; τ	λ	None
Manias 2014 [26]; β	34 (3)	To identify interventions that reduce MEs in pediatric ICUs	Pediatric	ICU only	Intervention	None	9; To 2014; τ	ϕ	Transcription error (0.56 was reported as 0.056) [27].
Nuckols 2014 [28]; β	16 (6)	To assess effectiveness of CPOE to reduce PADEs in hospital acute care settings	Adult	Acute care settings	Intervention evaluating CPOE vs. paper order	Screened on: peer-review; event detection method; specific event type or condition^d^	8; To 23 Sept 2013; μ	ϕ, λ, χ, ρ, ψ	None
Boeker 2013 [29]; β	6 (4)	To review the occurrence and nature of ADEs in surgical patients	Adult	Surgical settings only	Prospective studies	None	2; 1980–2011; π	ϕ, λ	None
Hakkarainen 2012 [30]; α	22 (8)	To estimate the percentage of patients with PADRs and the preventability of ADRs in adult outpatients and inpatients	Adult	All hospital settings (excluded if only ICU)	Any	Screened on: peer-review; event detection method; specific event type, condition, or treatment; preventability assessment; outcome of interest^e^	7; To Sept 2010; τ	ϕ, χ	One study included “potentially preventable events” as well as PADRs, inflating the PADR incidence [31]. Data for 3 other studies included PADRs acquired pre-admission as well as in hospital, inflating the PADR incidence [32–34].
Manias 2012 [35]; β	24 (6)	To identify interventions that reduce MEs in ICUs	No age restriction	ICU only	Intervention	Excluded if incidence of ME not reported	11; To Oct 2011; τ	ϕ, §	Reported “potential PADRs” instead of PADRs for one study, inflating the PADR incidence from 4 to 53.6 PADRs/1,000 patient-days [36].
Damiani 2009 [37]; θ	16 (1)	To assess impact of systematic safety processes on different ME categories	No age restriction	Not reported (assumed to be all hospital settings)	Any study quantitatively evaluating the impact of different systematic safety processes on error reduction	None	4; 1997–Apr 2007; μ	ρ	None
Hodgkinson 2006 [38]; θ	23 (1)	To assess strategies to reduce MEs in geriatrics in acute, subacute, and residential care settings	Geriatric	Acute, subacute, and residential care settings	Any (but focused on systematic reviews and RCTs)	None	13; 1986–Feb 2005; τ	ϕ, λ	None

^a^ Funding source: α = no funding source; β = non-profit or public funding source; θ = funding source not reported

^b^ Search strategy language restriction: τ = English only; π = all languages included; μ = no restriction reported

^c^ PADR incidence unit of measure: ϕ = PADRs/100 patients; λ = PADRs/1,000 patient-days; χ = percentage of patients experiencing at least one PADR; ρ = PADRs/1,000 doses; ψ = PADRs/1,000 medication orders; § = PADRs/1,000 opportunities for error

^d^ Included only peer-reviewed studies; excluded studies that did not report event detection method or that used voluntary reporting alone to detect events; excluded studies addressing events limited to specific conditions or types of errors

^e^ Included only peer-reviewed studies; excluded studies using voluntary reporting or ICD-9 or 10 codes to detect events; excluded studies representing specific disease areas, treatments, or types of ADRs; excluded if all dose-dependent and predictable ADRs were considered preventable without a separate preventability assessment; excluded if percentage of patients with PADRs or the preventability of ADRs was not reported

### Findings from evaluations of systematic review quality

All the included reviews had multiple flaws as per the AMSTAR-2 assessment tool (see S5 Text). Two reviews (Nuckols et al., 2014 [33] and Maaskant et al., 2015 [35]) had fewer flaws than the other reviews; however, neither of these reviews had estimation of PADR incidence as their review objective. Only one review [35] had registered a protocol a priori, while four others [27,29,32,33] had a written protocol or guide that was not registered. None of the reviews used a comprehensive literature search strategy, as defined by the AMSTAR-2 guidelines; however, all but one [28] achieved a “partial yes” response, indicating that they had searched at least two databases relevant to the search question, provided keywords or a search strategy, and justified publication restrictions such as language. Three reviews [25,33,35] provided a list of excluded studies and justified the exclusions. All three reviews that included randomized controlled trials (RCTs) used a satisfactory technique to assess risk of bias; however, assessment of risk of bias in non-randomized studies was less satisfactory (two of 13 studies used a satisfactory tool, while nine others achieved a “partial yes” response). AMSTAR-2 elements that were generally of high quality were use of PICO components in review questions and inclusion criteria, explanation of study designs for inclusion, use of appropriate methods for meta-analysis of RCTs (where conducted), and reporting of potential sources of conflict of interest.

### PADR incidence reported in systematic reviews

One review [32] conducted meta-analyses to estimate a pooled PADR incidence from individual patient data (IPD) from four primary studies, and produced an estimate of 4.9 PADRs per 100 patients (95% confidence interval: 4.3–5.6). Estimates of PADR incidence from at least one primary study were reported in the other 12 reviews and are summarized for each of the five reported units of measure in Table 4. Overall, the range of PADR incidence reported across all reviews for each unit of measure was wide (from 0.006 to 17 PADRs per 100 patients), demonstrating the substantial heterogeneity of the primary studies that were included in the reviews.

**Table 4:** PADR incidence reported in the 13 included systematic reviews.

Review first author/year	PADRs per 100 patients [primary study citations]	PADRs 1,000 patient days [primary study citations]	% of patients experiencing at least one PADR [primary study citations]	PADRs per 1,000 doses [primary study citations]	Other units of measure reported [primary study citations]
Kanagaratnam 2016 [13]			2.4^a^^,^^b^ (1.4–3.7) [15]; 30.7^c^ (19.0–44.7) [14]		
Boeker 2015 [16]	Pooled estimate: 4.9 (95% CI = 4.3–5.6) [17,39–41]; 2.6 [42]		3.9 [41]; 4.1 [40]; 5.2 [39]; 8.4^a^ [17]		
Maaskant 2015 [18]		0.1^d^ [19]; 7.9 [43]			
Salmasi 2015 [20]	0.006 [44]; 0.007 [21]				
Wang 2015 [22]		4 [36]; 14 [24]; 28.9^e^ [23]			
Acheampong 2014 [25]		4 [36]			
Manias 2014 [26]	0.1 [45]			0.86 [46]; 0.56^f^ [27]	
Nuckols 2014 [28]	0.5 [47]; 10.6 [48]	2.9 [24]; 4.5 [49]	15.5 [50]	0.137 [51]	14.3 PADRs/1,000 medication orders [50]
Boeker 2013 [29]	1.7 [52]; 3.6 [53]; 4.1 [41]; 5.9 [39]	3.3 [52]; 5.1 [53]			
Hakkarainen 2012 [30]	0.3^a^^,^^g^ [34]; 0.4^h^ [32]; 3d [31]; 8.8 [54]; 11.8^a^ [55]; 12.8^a^ [56]; 16.2 [57]; 17 [33]		0.3^a^^,^^g^ [34]; 8.6^a^ [33]		
Manias 2012 [35]	0.41 [58]	4.8 [59]; 10.7 [24]; 53.6^e^^,^^i^ [36]		0.137 [51]	0.6 PADRs/100 opportunities for error [60]
Damiani 2009 [37]				0.137 [51]	
Hodgkinson 2006 [38]	11.4 [61]	26.5 [61]			

^a^ Included PADRs that led to admission or caused by drugs given pre-admission (i.e., community-acquired PADRs) as well as in-hospital PADRs

^b^ Calculated from data presented in the review. Reported in primary paper as 33 PADRs/1,332 patients (67 patients experienced 69 ADRs of which 33 were preventable).

^c^ Included only PADRs that led to admission (i.e., community-acquired PADRs); also included “probable” and “likely” PADRs, as well as “definite”

^d^ It is unclear from the primary study if these are only PADRs or are all ADRs. Data from the pre-intervention and post-intervention periods were pooled.

^e^ Included potential PADRs (e.g., medication errors that did not cause harm) as well as actual PADRs

^f^ Transcription error: should have been reported as 0.056 PADRs/1,000 doses

^g^ Data for this study were reported in two different figures in the review, with different units but the same value

^h^ Unclear if some events occurred pre-admission

^i^ Actual PADR incidence was 4.0 PADRs/1,000 patient-days, as reported by Acheampong et al. [25] and Wang et al. [22]

The Jadad framework identified several differences in the included reviews that could account for the discordance in the PADR incidences reported (**Table 3**). Considerable heterogeneity was noted regarding review objectives, patient age of interest, setting of interest, other selection criteria of the primary studies and search strategies (and related date coverage), which resulted in minimal overlap of primary studies within the reviews and considerable differences in PADR incidences reported. Additionally, errors in the reporting of PADR data were noted in some reviews that resulted in rare but substantial inaccuracies in reported incidence. These errors included transcription errors in decimal point location; inclusion of “potential” PADRs (i.e., MEs that did not result in patient harm) as well as actual PADRs, which inflated PADR incidence; inclusion of community-acquired PADRs present at hospital admission as well as inpatient PADRs, which inflated PADR incidence; and possible mislabelling of ADRs reported in primary studies as PADRs in reviews.

### Secondary review questions—Findings from systematic reviews

One review [32] meta-analysed IPD from four studies and found that the incidence of PADRs was significantly lower in surgical versus non-surgical inpatients: 4.2 (95% CI: 3.5–5.1) versus 5.7 (95% CI: 4.8–6.8), respectively (p = 0.024). No other reported findings were relevant to the secondary review questions of interest.

### Primary study characteristics

Following full-text screening of the 37 primary studies for which in-hospital PADR data were reported in the set of included reviews, four studies were excluded: two because PADRs present at admission were included as well as in-hospital events [46,55], one because only PADRs present at admission were included (i.e., no in-hospital events) [68], and one because MEs that did not cause harm were included in the PADR definition [63]. An additional four studies were considered for inclusion that had not been included in the original overview of reviews. Two of these [22,75] had been identified in a review that was ultimately excluded because no raw PADR data had been reported [76], one had been reported in an included review [26] but had no usable PADR data reported for it in the related review [77], and one had originally been excluded because its review [33] had implied that the PADR data had included potential ADRs as well as PADRs (but inspection of the full text of the primary study confirmed only PADRs had been counted) [78]. In total, PADR incidence data were extracted from 37 primary studies [22,38–45,47–54,56,57,60–62,64–67,69–75,77–80]. Their characteristics have been summarized in Tables 1 and 2 and reported in detail in Table 5.

**Table 5:** Characteristics of the studies included in primary study syntheses.

Study first author/year; country; funding source^a^	Objective	Patient age category	Hospital setting/ discipline^b^	Event detection method	ADR definition	Causality assessment	Preventability assessment	PADR incidence units of measure^c^
Aljadhey 2013 [42]; Saudi Arabia; β	To assess the incidence of in-hospital ADEs, potential ADEs, and MEs	Adult	All wards/ ICU	Prospective daily chart review + stimulated reporting (MEs and ADRs)	NCC MERP categories E-I [3]	Brigham and Women’s Hospital [5]	All MEs considered preventable	ϕ, λ
de Boer 2013 [40]; The Netherlands; β	To assess the incidence and nature of ADEs and risk factors in surgical patients	Unclear	Surgical wards	Retrospective chart review with triggers (ADEs)	Injury from a drug	Custom [62]	Custom	ϕ
Laroche 2013 [15]; France; β	To assess the prevalence of ADRs in patients with dementia	Geriatric	Long-term care	Prospective one-time chart review for prevalence (ADRs)	WHO [1]	French causality assessment tool [6]	French Adverse Drug Reactions Preventability Scale [12]	ϕ
Leung 2012 [48]; USA; β	To assess the impact of vendor CPOE systems on the frequency of ADEs	Adult	All wards/ ICU	Retrospective chart review with triggers (ADEs)	Injury from a drug	Brigham and Women’s Hospital [5]	All MEs considered preventable	ϕ
Menendez 2012 [47]; Spain; θ	To assess the impact of an electronic clinical record on ME frequency and severity	Geriatric	Not reported	Voluntary reporting (MEs)	NCC MERP categories E-I [3]	Not reported	Not reported	ϕ, ρ
Abstoss 2011 [27]; USA; θ	To assess the impact of 4 cultural and 3 system-level interventions for medication safety in an ICU on ME rates	Pediatric	ICU only	Voluntary reporting (MEs)	NCC MERP categories E-I [3]	Not reported	All MEs considered preventable	ρ
Morimoto 2011 [41]; Japan; β, γ	To assess the incidence and preventability of ADEs and MEs in Japan	Adult	All wards/ ICU	Prospective daily chart review + voluntary reporting (MEs and ADEs)	Injury from a drug	Brigham and Women’s Hospital [5]	All MEs considered preventable	ϕ, λ
Chapuis 2010 [60]; France; β	To assess the impact of an automated dispensing system on the incidence of MEs in a medical ICU	Adult	Medical ICU	Other: Direct observation of picking, preparation, and administration of drugs, with intervention when MEs identified (MEs)	NCC MERP categories E-H [3]	Not reported	All MEs considered preventable	ϕ, §
Klopotowska 2010 [36]; The Netherlands; β	To assess the impact of hospital pharmacist participation on prescribing errors and PADEs in ICUs	Adult	ICU only	Other: Daily medication order review, with intervention when MEs identified (MEs)	NCC MERP categories E-I [3]	Not reported	All MEs considered preventable	ϕ, λ
Berga Cullere 2009 [39]; Spain; β	To assess the incidence and preventability of ADEs in hospitalized patients	Adult	Medical and surgical wards	Prospective daily chart review with triggers + daily team interview (ADRs)	Injury from a drug	Karch & Lasagna [63]	Adaptation of Schumock and Thornton [9]	ϕ
Davies 2009 [55]; UK; β	To assess the incidence of ADRs in in-patients, their impact on length of stay and costs, and their risk factors	Adult	Medical and surgical wards	Prospective daily chart + voluntary and stimulated reporting (ADRs)	Edwards and Aronson [2]	Naranjo [64]	Hallas [11]	ϕ
Morriss 2009 [46]; USA; β	To assess the impact of a barcode medication administration system on PADEs in the NICU	Pediatric	ICU only	Prospective daily chart review with triggers + voluntary reporting (MEs and ADEs)	Injury from a drug	Not reported	All MEs considered preventable	ϕ, λ, ρ
Pourseyed 2009 [54]; Iran; β	To assess the frequency and nature of ADRs as a cause for admission or when occurring after admission	All ages	Medical wards	Prospective daily chart review + daily patient interview (ADRs)	WHO [1]	WHO Probability Scale [2,8]	Adaptation of Schumock and Thornton [9]	ϕ
Van Doormaal 2009 [50]; The Netherlands; β	To assess the impacts of CPOE/CDSS on the incidence of MEs and patient harm	Adult	Medical wards	Prospective chart review (MEs)	NCC MERP categories E-H [3]	Custom	All MEs considered preventable	χ
Baniasadi 2008 [32]; Iran; θ	To assess the incidence and nature of ADRs in a newly established ADR reporting centre	All ages	All wards/ ICU	Voluntary reporting (ADRs)	WHO [1]	Naranjo [64]	Schumock and Thornton [9]	ϕ
Gurwitz 2008 [65]; Canada & USA; θ	To assess the impact of CPOE/CDSS on PADEs in long-term care	Geriatric	Long-term care	Retrospective chart review with triggers (ADEs)	Injury from a drug	Custom	All MEs considered preventable	λ
Handler 2008 [66]; USA; β, γ	To assess the incidence and positive predictive values of triggers to detect ADRs in a nursing home	Geriatric	Long-term care	Retrospective chart review with triggers (ADRs)	WHO [1]	Naranjo [64]	All MEs considered preventable	ϕ
Nuckols 2008 [59]; USA; γ	To assess the impact of smart IV pumps compared to conventional IV pumps on the incidence of PADEs	Adult	Medical and surgical ICU	Retrospective chart review with triggers (ADEs)	Injury from a drug	Not reported	Dubois and Brook [10]	ϕ, λ
Walsh 2008 [43]; USA; β	To assess the impact of a commercial CPOE system on the incidence of non-intercepted serious MEs in pediatrics	Pediatric	All wards/ ICU	Retrospective chart review (MEs and ADEs)	Injury from a drug	Not reported	Custom	ϕ, λ
Weant 2007 [51]; USA; θ	To assess the impact of a CPOE on the incidence and type of MEs	Unclear	Neurosurgical ICU	Voluntary reporting (MEs)	NCC MERP categories E-H [3]	Not reported	Not reported	ρ
Bradley 2006 [58]; USA; θ	To assess the impact of a CPOE/CDSS on the rate and nature of reported MEs	Unclear	All wards/ ICU	Voluntary and stimulated reporting (MEs)	NCC MERP categories E-H [3]	Not reported	All MEs considered preventable	ϕ, ρ
Colpaert 2006 [67]; Belgium; θ	To assess the impact of a CPOE/CDSS on the incidence and severity of prescribing errors in an ICU	Unclear	Surgical ICU	Retrospective medication order review^d^ (MEs)	NCC MERP categories D-I [3]	Not reported	Not reported	ϕ, λ, ψ
Davies 2006 [56]; UK; β	To develop a methodology and assess its feasibility to estimate the burden of in-patient ADRs	Adult	Medical and surgical wards	Prospective daily chart review with triggers + voluntary and stimulated reporting (ADRs)	Edwards and Aronson [2]	Naranjo [64]	Hallas [11]	ϕ
Hintong 2005 [21]; Thailand; β	To assess the nature, contributing factors and preventive strategies of MEs during anaesthesia	Unclear	Anaesthesia only	Voluntary reporting (MEs)	Custom	Not reported	All MEs considered preventable	ϕ
Cohen 2004 [68]; USA; θ	To assess the impact of the medication safety component of a patient safety program on the harm caused to patients by MEs	Unclear	All wards/ ICU	Retrospective chart review with triggers (ADRs)	NCC MERP categories F-I [3]	Not reported	Not reported	ϕ, λ, ρ
Dormann 2004 [33]; Germany; β	To assess if ADRs are predictors for recurrent hospitalizations in internal medicine	Unclear	Medical wards	Prospective daily chart review + daily patient monitoring (ADRs)	WHO [1]	Naranjo [64]	Schumock and Thornton [9]	χ
King 2003 [19]; Canada; θ	To assess the impact of a commercial CPOE system on MEs and ADEs in pediatric inpatients	Pediatric	Medical and surgical wards	Voluntary reporting (MEs)	Injury from a drug	Not reported	All MEs considered preventable	ϕ, λ
Kucukarslan 2003 [61]; USA; θ	To assess the impact of pharmacist participation in a physician rounding team on PADEs in general medicine units	Unclear	Medical wards	Retrospective chart review to detect ADEs (unclear) (PADEs)	Not reported	Not reported	Custom	ϕ, λ
Sangtawesin 2003 [44]; Thailand; θ	To assess the incidence and nature of MEs, severity of events, patient outcomes, and drug categories involved over a 15-month period in a pediatric hospital	Pediatric	All wards/ ICU	Voluntary reporting (MEs)	Custom	Not reported	Not reported	ϕ
Mullett 2001 [45]; USA; β	To assess the impact of an anti-infective decision support tool in a pediatric ICU	Pediatric	ICU only	Other: Computerized alerting programs reported mismatches of (1) culture and sensitivity results with patient antibiotic therapy and (2) anti-infective dosages with published therapeutic ranges. (MEs and ADEs)	Not reported	Not reported	Not reported	ϕ
Bates 1999 [69]; USA; β	To assess the impact of a CPOE on the incidence and nature of MEs	Unclear	Medical ICU and wards	Prospective daily chart review + voluntary and stimulated reporting (MEs and ADEs)	Injury from a drug	Not reported	Custom [52]	ϕ, λ, ψ
Gholami 1999 [57]; Iran; θ	To assess the incidence of ADRs in in-patients and the factors associated with preventability, predictability, and severity of ADRs	Adult	Medical wards	Prospective daily chart review and lab monitoring + daily patient interview (ADRs)	WHO [1]	Naranjo [64]	Adaptation of Schumock and Thornton [9]	ϕ
Leape1999 [24]; USA; β	To assess the impact of pharmacist participation on medical rounds in the ICU on the rate of PADEs caused by ordering errors	Unclear	Medical ICU and CCU	Retrospective chart review (MEs and ADEs)	Injury from a drug	Not reported	Dubois and Brook [10]	ϕ, λ
Bates 1998 [49]; USA; β	To assess the impact of a CPOE and a team-based intervention on non-intercepted serious MEs	Adult	All wards/ ICU	Prospective daily chart review + voluntary and stimulated reporting (ADEs)	Not reported	Not reported	Dubois and Brook [10]	ϕ, λ
Bates 1995 [52]; USA; Β	To assess the incidence and preventability of ADEs and potential ADEs	Adult	All wards/ ICU	Prospective daily chart review + voluntary and stimulated reporting (ADEs)	Injury from a drug	Not reported	Dubois and Brook [10]	ϕ, λ
Pearson 1994 [34]; USA; γ	To assess factors associated with PADRs in community hospital patients, to characterize PADRs, and to assess the impact of PADRs on length of stay	Adult	All wards/ ICU	Voluntary reporting + possibly prospective patient monitoring (ADRs)	WHO [1]	Naranjo [64]	Adaptation of Schumock and Thornton [9]	ϕ
Bates 1993 [53]; USA; β	To assess the incidence and preventability of ADEs, the incidence of potential ADEs and the number actually prevented, and the yield of strategies to detect ADEs and potential ADEs	Adult	All wards/ ICU	Prospective daily chart review + voluntary and stimulated reporting (MEs and ADEs)	WHO [1]	Naranjo [64]	Dubois and Brook [10]	ϕ, λ

^a^ Funding source: α = no funding source; β = non-profit or public funding source; γ = industry funding; θ = funding source not reported

^b^ The discipline of studies set in “all wards/ICU” or “ICU only” was categorized as “Medicine and surgery.” Long-term care was categorized as “Medicine.”

^c^ PADR incidence units of measure: ϕ = PADRs/100 patients; λ = PADRs/1,000 patient-days; χ = percentage of patients experiencing at least one PADR; ρ = PADRs/1,000 doses; ψ = PADRs/1,000 medication orders; § = PADRs/1,000 opportunities for error

^d^ The appropriateness of the drug was not considered in the detection of medication errors

The 37 included primary studies were published between 1993 and 2013, with a variety of objectives, using both interventional and other study designs [22,38–45,47–54,56,57,60–62,64–67,69–75,77–80]. There was high heterogeneity of patient age, hospital setting, medical discipline, and endpoint of interest. More than half of the studies (59%) relied upon non-prospective methods of event detection [22,38,39,43,47–49,52,54,56,61,62,64–67,70,71,73,75,77,78]. The ADR definition was highly varied—12 studies (32%) used “injury from a drug,” eight (22%) used the WHO definition [6], 10 (27%) used one of four different category groupings of the National Coordinating Council for Medication Error Reporting and Prevention (NCC MERP) Index [81], two (5%) used the definition from Edwards and Aronson (2000) [82], two (5%) developed custom definitions, and three (8%) did not report a definition. Causality assessments were reported in only 18 (49%) of the included studies. Many of these causality assessments were conducted using the Naranjo tool [83] (8 of 18 studies; 44%), although three recent studies with co-authorship at Brigham and Women’s Hospital (Boston, USA) reported using a tool developed at that institution [57,62,74]. Preventability assessments were conducted in 84% of studies, with the concept that “all medication errors are preventable” being the most frequently cited (n = 14; 38% of all studies). Sample size was also highly varied, ranging from 79 to 202,699 patients.

#### PADR incidence

**Table 6** presents the PADR incidence rates calculated from raw data reported in the primary studies. The most common unit of measure was the number of PADRs occurring in a given number of patients, from which PADRs per 100 patients could be calculated (32 of 37 studies; 86%) [22,38–40,42–45,47–53,56,57,61,62,64–67,69,70,72–74,77–80]. These data formed the basis of our syntheses and statistical analyses. Sixteen studies (43%) reported data from which PADRs per 1,000 patient-days could be calculated (see S6 Text) [38,48,50,51,53,57,64,65,70,73–75,77–80].

**Table 6:** PADR incidence^a^ reported in the primary studies.

Study first author/year; country	Event detection method	Total number of patients (patient-days)	PADRs per 100 patients	PADRs per 1,000 patient days	% of patients experiencing at least one PADR	PADRs per 1,000 doses	Other units of measure reported
Sangtawesin 2003 [44]; Thailand	Voluntary reporting (MEs)	32,105	0.006^b^,^c^				
Hintong 2005 [21]; Thailand	Voluntary reporting (MEs)	202,699	0.007^b^				
King 2003 [19]; Canada	Voluntary reporting (MEs)	30,317 (140,897)	0.06^b^^,^^d^	0.1^b^^,^^d^			
Mullett 2001 [45]; USA	Other: see Table 5 (MEs and ADEs)	487	0.2				
Pearson 1994 [34]; USA	Voluntary reporting + possibly prospective patient monitoring (ADRs)	10,587	0.3^b^^,^^e^				
Leung 2012 [48]; USA	Retrospective chart review with triggers (ADEs)	30,161	0.4				
Baniasadi 2008 [32]; Iran	Voluntary reporting (ADRs)	6,840	0.4^b^^,^^f^				
Bradley 2006 [58]; USA	Voluntary and stimulated reporting (MEs)	2,450	0.4^b^			0.09	
Menendez 2012 [47]; Spain	Voluntary reporting (MEs)	7,001	0.5^b^			0.04	
Bates 1999 [69]; USA	Prospective daily chart review + voluntary and stimulated reporting (MEs and ADEs)	379 (1,704)	1.4	2.9			0.5 PADRs/1,000 medication orders
Klopotowska 2010 [36]; The Netherlands	Other: Daily medication order review, with intervention when MEs identified (MEs)	115	1.7^c^	4.0^c^			
Bates 1995 [52]; USA	Prospective daily chart review + voluntary and stimulated reporting (ADEs)	4,031 (21,412)	1.7	3.3			
Nuckols 2008 [59]; USA	Retrospective chart review with triggers (ADEs)	4,604 (20,559)	2.2^g^	4.9^g^			
Bates 1998 [49]; USA	Prospective daily chart review + voluntary and stimulated reporting (ADEs)	2,491 (12,218)	2.2	4.5			
Laroche 2013 [15]; France	Prospective one-time chart review for prevalence (ADRs)	1,332	2.5^e^^,^^h^				
Aljadhey 2013 [42]; Saudi Arabia	Prospective daily chart review + stimulated reporting (MEs and ADRs)	977	2.6	2.6			
Chapuis 2010 [60]; France	Other: Direct observation of picking, preparation, and administration of drugs, with intervention when MEs identified (MEs)	1,001	3.5				5.7 PADRs/1,000 opportunities for error
Bates 1993 [53]; USA	Prospective daily chart review + voluntary and stimulated reporting (MEs and ADEs)	420 (2,967)	3.6	5.1			
Walsh 2008 [43]; USA	Retrospective chart review (MEs and ADEs)	275 (1,368)	4.0	7.9			
Morimoto 2011 [41]; Japan	Prospective daily chart review + voluntary reporting (MEs and ADEs)	3,459 (59,383)	4.1	2.4			
de Boer 2013 [40]; The Netherlands	Retrospective chart review with triggers (ADEs)	567	4.2				
Handler 2008[66]; USA	Retrospective chart review with triggers (ADRs)	274	5.8				
Berga Cullere 2009 [39]; Spain	Prospective daily chart review with triggers + daily team interview (ADRs)	1,550	5.9		5.3		
Gholami 1999 [57]; Iran	Prospective daily chart review and lab monitoring + daily patient interview (ADRs)	370	8.1				
Morriss 2009 [46]; USA	Prospective daily chart review with triggers + voluntary reporting (MEs and ADEs)	475 (6,094)	8.2	6.5		0.86	
Cohen 2004 [68]; USA	Retrospective chart review with triggers (ADRs)	120 (730)	8.3	13.7		0.59	
Pourseyed 2009 [54]; Iran	Prospective daily chart review + daily patient interview (ADRs)	400	8.8				
Leape 1999 [24]; USA	Retrospective chart review (MEs and ADEs)	225 (1,892)	10.7^i^	12.7^i^			
Kucukarslan 2003 [61]; USA	Retrospective chart review to detect ADEs (unclear) (PADEs)	79 (339)	11.4	26.5			
Davies 2009 [55]; UK	Prospective daily chart + voluntary and stimulated reporting (ADRs)	3,322	11.8^e^				
Davies 2006 [56]; UK	Prospective daily chart review with triggers + voluntary and stimulated reporting (ADRs)	125	12.8^e^				
Colpaert 2006 [67]; Belgium	Retrospective medication order review (MEs)	90 (80)	13.3^j^	150^j^			10.0^j^ PADRs/1,000 medication orders
Abstoss 2011 [27]; USA	Voluntary reporting (MEs)	Not reported				0.06	
Van Doormaal 2009 [50]; The Netherlands	Prospective chart review (MEs)	592			15.5		
Gurwitz 2008 [65]; Canada & USA	Retrospective chart review with triggers (ADEs)	Not reported (97,710)		1.3			
Weant 2007 [51]; USA	Voluntary reporting (MEs)	Not reported				0.14	
Dormann 2004 [33]; Germany	Prospective daily chart review + daily patient monitoring (ADRs)	844			7.3^e^		

^a^ Raw data have been converted to rates and rates have been reported in common units.

^b^ Events detected by voluntary or stimulated reporting only

^c^ PADRs were prevented after error detection, if possible, reducing PADR rate

^d^ It is unclear if these are only PADRs or are all ADRs

^e^ Included PADRs that led to admission or caused by drugs given pre-admission (i.e., community-acquired PADRs) as well as in-hospital PADRs

^f^ Unclear if some events occurred pre-admission

^g^ only PADEs related to IV drugs in ICU infusion pumps

^h^ Prevalence study, not incidence

^i^ Only PADRs preventable at the drug ordering stage

^j^ Included some MEs that did not cause harm but required increased patient monitoring or intervention (i.e., not PADRs)

**Fig 3:**
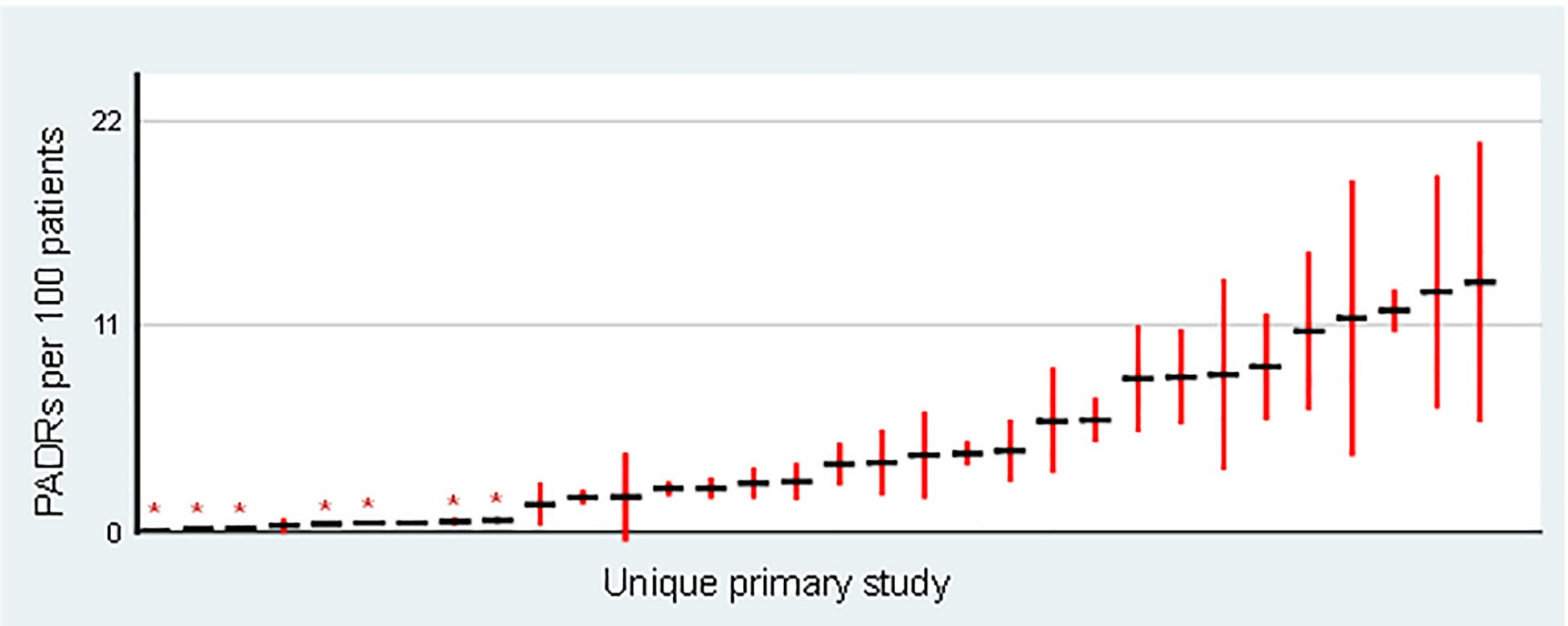
Scatterplot of PADR incidence rates calculated from raw data reported in primary studies, with 95% confidence intervals. Asterisks (*) indicate studies using voluntary/stimulated reporting alone as the event detection method. Point estimates of the PADR incidence rate for each study are represented by horizontal black lines, with their 95% confidence intervals represented by vertical red lines.

**Fig 4:**
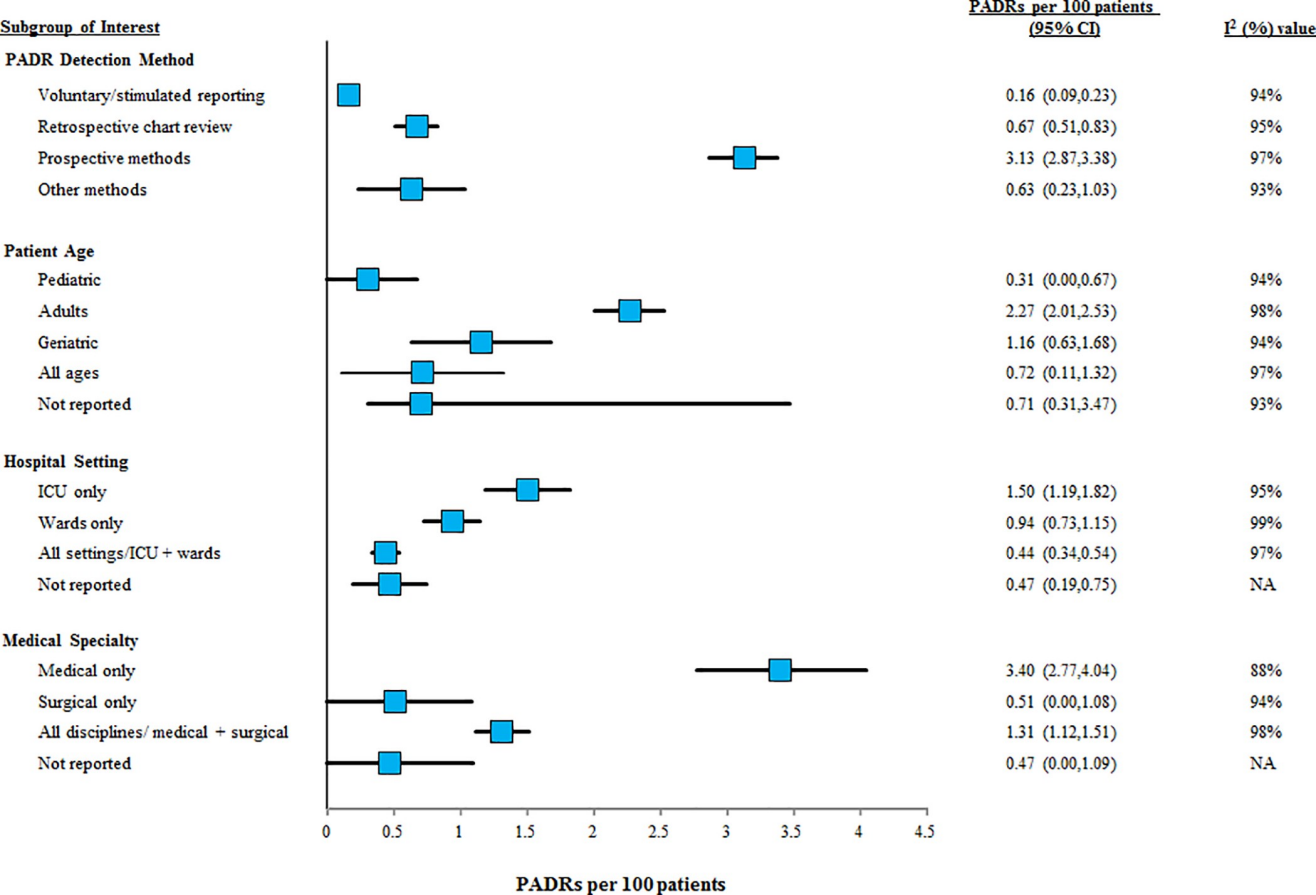
Subgroup meta-analyses, PADR incidence from primary studies. Pooled incidence rates of PADRs per 100 patients are reported with 95% confidence intervals. Measures of statistical heterogeneity (I2) are reported alongside each meta-analysis.

PADR incidence varied substantially among the 32 studies (range: 0.006 to 13.3 PADRs per 100 patients) (Fig 3). The three highest PADR incidences were reported in studies with designs that may inflate event count: one study [78] (13.3 PADRs per 100 patients) included events that caused increased patient monitoring but no harm as well as those that caused patient harm, while the other two studies [42,45] included some PADRs caused by drugs started prior to admission as well as inpatient PADRs (11.8 and 12.8 PADRs per 100 patients). Visual exploration of the data suggested a potential association between voluntary/stimulated reporting as the sole method of event detection and low PADR incidence rates (Fig 3). Subgroup meta-analysis demonstrated that PADR incidence varied significantly between the four categories of event detection methods (Fig 4), with studies using voluntary/stimulated reporting being associated with significantly lower PADR incidence than all other event detection methods. The true PADR incidence rate in the population was estimated as 3.13 PADRs per 100 patients (95% CI: 2.87–3.38) from the 13 studies using prospective methods of event detection. However, heterogeneity was extremely high across these 13 studies (I^2^ = 97%), suggesting that the pooled PADR incidence reported should be interpreted with caution.

In addition to the main subgroup meta-analyses presented below, subgroup meta-analyses demonstrating the influence of event type of interest, ADR/ADE definition, causality assessment tool, and preventability assessment tool used in the primary studies on PADR incidence are presented in S6 Text.

#### The influence of patient age

Two studies reported an assessment of the impact of patient age on PADR incidence. In a sample of patients mostly aged between 60–80 years (mean 66.317.90 (SD) years) in non-ICU hospital settings, Berga Culler et al. [72] determined that patients with PADRs were 5.4 years older (95% CI: 0.1–10.7) than patients with non-preventable ADRs. In a study by de Boer et al [56], the PADR incidence in patients aged >65 years was significantly higher than in younger adults aged 17–65 years (incidence rate ratio = 2.77; 95% CI: 1.14–6.72).

In our subgroup meta-analysis of patient age, the pooled PADR incidence estimate of adult studies was significantly higher than the pooled estimates in all other patient age categories (Fig 4). However, the number of studies in the other age categories was low and the heterogeneity was extremely high between studies (I^2^ = 98%) and within all age groups (I^2^ range: 92–98%).

#### The influence of hospital setting

No study explicitly discussed the influence of hospital setting on PADR incidence. Our subgroup meta-analysis found that compared to ICU settings, all other unit types had significantly lower PADR incidence (Fig 4). There was substantial statistical heterogeneity between studies (I^2^ = 98%) and within all age groups (I^2^ range: 95–99%).

#### The influence of medical specialty

One study commented specifically on the influence of medical specialty on PADR incidence: the proportion of ADRs that were preventable was 50% vs 53.6% in medical wards and surgical wards, respectively [72]. No test of significance was performed.

Our subgroup meta-analysis of medical specialty demonstrated that, compared to medical patients alone, all other discipline groups had significantly lower PADR incidence (Fig 4). There was extreme statistical heterogeneity between studies (I^2^ = 98%) and within all subgroups (I^2^ range: 88–98%).

#### Types of preventable events

One study provided insight specifically into the influence of medication error/drug-level factors on PADR incidence. Bates et al. [50] reported that 93% (14 of 15) of the PADRs that they identified were “Type A,” that is, dose-dependent, related to the pharmacologic characteristics of the drug, and predictable (e.g., a patient receiving the incorrect dosage of hydromorphone for pain control, resulting in either uncontrolled pain or side effects such as reduced level of consciousness). The other PADR detected was “Type B” and due to a patient receiving a drug to which they had a known allergy.

Almost half of the included studies provided data on the stage of the medication process at which errors occurred that resulted in PADRs, but none reported definitions of the stages reported (see detailed table provided in S7 Text). Thirteen medication process stages were reported in the literature, with eight stages being reported in three or fewer studies. The proportion of PADRs associated with any one stage in any given study was dependent upon the number and types of other stages reported in the study. Medication process stages reported in some studies overlapped with two or more stages reported in others, and this influenced the proportion of PADRs reported. For example, regarding ordering errors, one study [57] measured only ordering errors (96% of PADRs) and dispensing errors (4%), while another study [72] did not report “ordering errors” and instead differentiated between omission of a dose/medication (36%), wrong dose (29%), wrong medication (17%), inappropriate medication (14%), unnecessary medication (2%), etc. Because of the non-standardized medication process definitions, synthesis of these data could not be conducted. Two studies [48,74] reported data for the same three ME stages: ordering errors (51% and 34%, respectively), administration errors (14% and 11%, respectively), and insufficient monitoring (35% and 55%, respectively). No other study combinations could be compared. Generally, when they were reported, drug errors such as omission of a drug/medication and wrong drug accounted for a relatively high proportion of PADRs (seven studies; median 50%; range 29–57%); however, it could not be ascertained whether these drug errors occurred at the ordering, dispensing, or administration stages.

#### System-level causes of PADRs

Twenty intervention studies were categorized to summarize the effects of five system-level interventions on PADR incidence [38,47,48,49,52,53,54,60,61,62,64,65,70,71,73, 75,77,78,79,80]. Overall there was no consistent effect of any of the interventions on PADR incidence, other than cultural interventions (i.e., changing the systemic institutional attitude toward medication errors and adverse events). The two studies that evaluated a cultural intervention found a significant decrease in PADR incidence associated with implementation [54,77]; however, these cultural interventions were also combined with other interventions. The cultural interventions studied included (1) the implementation of a medication safety program in a community hospital that included intensive work on cultural change to increase ME reporting as well as the introduction of a number of drug protocols and standardized procedures, and (2) the implementation of four cultural interventions (poster tracking “days since last ME resulting in harm,” a continuous slideshow of performance metrics, didactic curricula, and emails summarizing MEs) as well as three system-level interventions (CPOE, pharmacist participation, and patient safety report form streamlining). A table provided in S8 Text summarizes the observed importance of each intervention’s effects on PADR incidence.

#### Severity of patient outcomes associated with PADRs

Fourteen studies [22,39,49,51,54,56,60,62,66,72,73,75,79,80] discussed the severity of harm of the PADRs detected, using numerous severity rating scales; a detailed table summarizing study specific findings is provided in S9 Text. The heterogeneity in the scales used precluded synthesis of the data; however, the proportions of PADRs detected at each severity level of the scale used in each study are presented in the table noted above. Overall, 20% or less of the PADRs detected tended to be in the higher severity categories (e.g., life-threatening or fatal). When the NCC MERP scheme [81] was used [49,54,60,72], most PADRs fell within the lowest severity category. For other scales, there appeared to be more PADRs of moderate severity than PADRs of extremely low or high severity.

#### Influence of drug class

Six studies commented on the risk of PADRs with respect to various drug classes [48,51,57,69,74,80]. Five of the six studies identified central nervous system depressants (sedatives or antipsychotics) as drugs associated with an increased risk of PADRs [48,51,69,74,80]. Other drugs identified as associated with PADRs by at least one study included antibiotics, anti-hypertensives, diuretics, NSAIDs, electrolytes, analgesics, and insulin.

Ten studies provided a breakdown of detected PADRs by drug class [39,51,56,57,61,62,72,74,75,80]; however, a standardized classification of drugs was not used across studies, and in some studies, more than one drug class could be attributed to a single PADR. Drug class data could not be synthesized but have been summarized in Table 7. Sedatives, anticoagulants, and antibiotics were the most frequently reported drug classes (n = 8 studies each). Cardiovascular drugs and analgesics were associated with the highest median proportion of PADRs, causing 18% (range: 1–28%) and 16% (range: 1–29%) of PADRs, respectively, in seven and five studies, respectively.

**Table 7:** The influence of drug class on PADR incidence.

**Drug class**	**% of PADRs caused by drug (median of studies reporting data)**	**Range of all studies reporting data**	**Number of studies; [primary study citations]**
Cardiovascular drugs	18%	1–28%	7 [34,39–41,48,52,65]
Analgesics	16%	1–29%	5 [34,39,41,48,52]
Anticoagulants	12.5%	3–29%	8 [34,39–42,48,52,65]
Opioids	11.5%	7–16%	4 [39,40,48,65]
Antibiotics/anti-infectives	11%	3–53%	8 [34,39–42,48,52,65]
Antihypertensives	11%	6–16%	2 [41,42]
Diuretics	10%	3–18%	5 [39,41,42,48,65]
Sedatives/anaesthetics	9%	1–80%	8 [34,39–41,48,52,65,69]
Antipsychotics	7%	2–50%	5 [15,41,48,52,65]
NSAIDs	7%	1–18%	4 [39,41,42,48]
Antidiabetics (oral and insulin)	5%	1–8%	4 [41,42,48,52]
Electrolytes/fluids	5%	1–18%	5 [39–41,48,52]
Hormonal drugs (excluding insulin and sex hormones)	4%	1–4%	3 [40,48,65]
Alimentary tract and metabolism drugs	3.5%	2–13%	6 [34,39–41,48,65]
Antiepileptics	3%	3–7%	3 [34,39,65]
Antineoplastics	3%	2–4%	2 [41,52]
Respiratory drugs	2%	1–3%	3 [34,48,65]
Antidepressants	1%	1–7%	3 [41,48,65]
Other drugs^a^	15%	4–23%	6 [34,39,40,48,52,65]
**Comments regarding the incidence of PADRs with respect to various drug classes found in the included primary studies**			
**Study**	**Comment**		
Aljadhey 2013 [42]	Antibiotics, antihypertensives, diuretics, and NSAIDs were the classes most frequently associated with PADEs, whereas anticoagulants were the drug most frequently associated with non-preventable ADEs		
Laroche 2013 [15]	Anti-dementia and antipsychotic drugs induced half of the ADRs of which most of them were preventable (dementia patients in various long-term care homes, units, and hospitals)		
Morimoto 2011 [41]	Sedatives, NSAIDs, and electrolytes were the classes most frequently associated with PADEs, whereas antibiotics were the class most frequently associated with non-preventable ADEs		
Nuckol 2008 [59]	In a study of IV drugs, half of PADRs occurred due to continuous infusions and 40% due to boluses. (morphine, insulin, fentanyl, and Propofol represented 44% of all drugs involved)		
Bates 1999 [69]	80% of PADRs in one of the study periods were due to the use of multiple sedating drugs (study set in medical ICUs and wards)		
Bates 1995 [52]	Antibiotics caused only 9% of PADEs versus 30% of non-preventable ADEs (p < 0.005). Central nervous system depressants, including sedatives and antipsychotics, were associated with PADEs more often than non-preventable ADEs. Analgesics were the most frequent PADE (29% of PADEs).		

^a^ “Other drugs” included undefined steroids, local anaesthetics, parenteral nutrition, allopurinol, calcium polystyrene, nutrients/supplements, hypoglycemics, muscle relaxants, gout medications, antihistamines, anti-Parkinson’s medications, and not reported drugs.

## Discussion

Spontaneous adverse drug reaction reporting programs provide important information on adverse events that may not have been detected during the pre-market clinical trial process. However, spontaneous reporting is limited by factors such as under-reporting and a lack of data on the population exposed to the drug that precludes the estimation of incidence rates. Research conducted outside of this system is necessary to acquire a better understanding of the occurrence of PADRs in specific populations or settings, such as hospitals. With this objective in mind, we undertook an overview of reviews, identifying 13 systematic reviews that reported in-hospital PADR data from 37 primary studies. However, obtaining a summary estimate of PADR incidence that could be generalized to all populations and settings was not possible due to heterogeneity of several sources within the primary studies. These included intra-study variability in the elements of the PADR definition, patient age, hospital setting, medical discipline, event detection methods, and others.

The definition of PADRs can be distilled into three concepts: (1) the underlying ADR or ADE definition, (2) the criteria used to assess causality, and (3) the criteria used to assess preventability. Across the published literature, considerable heterogeneity has been found in all 3 of these concepts [19,84,85], which was also present in our sample of primary studies. The ADR/ADE definition used by researchers can have considerable impact on the number of events ultimately identified as PADRs. When Bates et al. [50] used the WHO definition of ADR to reclassify 27 ADEs that they had originally identified using the definition “injury from a drug,” 12 of the events were excluded (44%), all of which had been judged to be preventable. The differences in ADR and ADE definitions are subtle but substantive. The WHO defines an ADR as “a response to a medicinal product which is *noxious and unintended* and which occurs *at doses normally used in man* for the prophylaxis, diagnosis or therapy of disease or for the restoration, correction or modification of physiological function” [6]. An ADE has been defined as “*any injury* occurring during the patient’s drug therapy and *resulting either from appropriate care*, *or from unsuitable or suboptimal care*. Adverse drug events include: the adverse drug reactions during normal use of the medicine, and any harm secondary to a medication error, both errors of omission or commission. An adverse drug event can result in different outcomes, notably: in the worsening of an existing pathology, in the lack of any expected health status improvement, in the outbreak of a new or to be prevented pathology, in the change of an organic function, or in a noxious response due to the medicine taken” [5]. ADR definitions are generally narrower than ADE definitions in that (1) they require the patient’s reaction to be noxious or harmful, whereas ADEs may be less injurious (e.g., simply the lack of any expected improvement); and (2) they require the dosage at which the reaction occurs to be normal use, whereas ADEs may occur at any dosage, including over- or under-dosage. Heterogeneity of the definition of ADR/ADE has resulted in some systematic reviews electing to include only primary studies that use a common definition [20,86]. For example, in an attempt to reduce heterogeneity, Miguel et al. [20] restricted inclusion to studies that used the WHO [6] definition or that of Edwards and Aronson [82]. In our review, eight different ADR/ADE definitions were employed in the 37 included studies. This extreme methodological heterogeneity undoubtedly contributed in part to the extremely high statistical heterogeneity found in all our meta-analyses.

Assessment of causality evaluates the likelihood of an adverse event being caused by a given treatment. Causality assessment allows the researcher to classify the likelihood of ADRs (e.g., uncertain, possible, probable, or certain) and to decrease disagreement between assessors in identification of ADRs [87]. Studies that use no causality assessment or that rely on informal assessment may have artificially inflated numbers of ADRs/PADRs, if events are included that would have been classified as non-drug-related when assessed using more formal and objective criteria. Commonly used tools include the Naranjo criteria [83], the French method [88], and the WHO-Uppsala Monitoring Centre tool [87]. Agreement between tools appears to be variable [89,90], and problems with reproducibility and validity have prevented the uptake of a universally accepted method [91]. In a review by Alhawassi et al. [84], half of all primary studies used no causality assessment tool, which is similar to our findings—19 of 37 studies (51%) did not assess causality. Heterogeneity of causality assessment tools across primary studies may increase the variability of PADR incidence estimates.

Preventability assessment determines whether an ADR could have been prevented. Instruments to assess preventability vary widely, with 18 unique tools identified in 143 primary studies reviewed by Hakkarainen et al. [85]. A systematic review in 2010 identified eight different approaches used in the primary literature to define “preventability;” it determined that none of the definitions fit all circumstances and that the reliability of the definitions was imperfect [4]. Thus, the accuracy of estimates of the preventability of ADRs was called into question. In our sample of primary studies, five different recognized tools and four other custom tools were used to assess preventability. Heterogeneity of preventability assessment tools across studies and the inherent inaccuracy of the tools themselves may lead to substantial differences in the events identified as PADRs and, ultimately, PADR incidence estimates.

The method used to detect events, whether they be MEs or ADRs/ADEs, has a significant impact on the number of events found and, consequently, the estimated incidence of events [8,92]. Voluntary submission of individual case reports to national regulatory authorities or medical journals is one of the foundations of drug safety surveillance and is a powerful first-line method to identify unanticipated effects of drugs [93]. However, voluntary reporting significantly underestimates the incidence of drug-related events due to under-reporting [19]. We found PADR incidence to be significantly lower in studies that used voluntary reporting methods than in studies that used any other detection method. As well in our review, studies of larger sample sizes were significantly more likely to employ voluntary reporting—its relatively low cost and limited personnel requirements likely contributing to its use in large studies. Given that prospective methods of event detection generally are thought to have the highest sensitivity to detect events (i.e., they detect a greater number of potential events) [18–20], we proposed that the pooled PADR incidence estimate of studies using prospective methods would most accurately reflect the true PADR incidence. However, considerable methodological and statistical heterogeneity present amongst the 13 studies employing prospective methods calls into question the validity of combining the studies and the generalizability of their pooled estimate. Given the low number of studies available, we were unable to perform meta-regression to adjust for multiple covariates at the same time.

Several of our secondary review questions were addressed through synthesis of data from the included primary studies. Our findings with respect to medical specialty and hospital setting reflected what has been previously published—namely that surgical patients experience a lower risk of PADRs than non-surgical patients [32] and that PADRs are less likely to occur in wards than in critical care units [57]. Surgical patients generally are younger, have elective procedures that are non-urgent and less complex, and have shorter hospital stays than medical patients, all of which contribute to fewer mediations administered and a lower risk of PADRs [32]. Similarly, ICU patients tend to be more complex cases than those in wards and require a greater number of drugs administered, increasing PADR risk [19]. While we found that ICU patients experienced a higher incidence of PADRs than ward patients (in concordance with the literature), it should be noted that our pooled estimate for the “ICU+Wards” group did not fall between the pooled estimates for the “ICU” and “Wards” groups and instead was significantly lower than both, indicating that high heterogeneity impacted these results.

With respect to patient age, the presence of multiple complex medical problems and polypharmacy in geriatric patients inherently elevates their risk of PADRs [18]. Compared to adults, pediatric patients may be expected to have a higher risk of ADRs because they do not have fully developed metabolic enzymes to clear drugs, and many of the youngest patients have low body fat, resulting in higher circulating levels of lipid-soluble drugs [18]. Pharmacokinetics of pediatrics change with age, sometimes within weeks in pre-term infants. Thus, pediatric dosing may require more calculations, which increases the chance of arithmetic error. Additionally, pediatric-specific dosage data are often lacking, forcing clinicians to use adult dosages [18]. However, others contend that fewer ADRs in pediatrics are preventable because they are less likely to be secondary to poor drug metabolism, which should be recognized, leading to adjustment in drug dosages [52]. Our review findings with respect to patient age were not clear cut due in part to overlapping patient age groups. The “adult” age group was associated with a significantly higher pooled PADR incidence rate than all other age groups; however, some of the studies contained in this group included a high proportion of geriatric patients, which may have influenced the findings. Additionally, none of the 12 “adult” studies in our subgroup analysis used voluntary event detection methods (9 used prospective methods), whereas the largest of the three “geriatric” studies used voluntary methods, likely weighting this subgroup negatively in the meta-analysis. Our included studies of pediatric patients tended to have a lower pooled PADR incidence than studies of adults or geriatrics; however, two studies that used voluntary reporting methods dominated the weighting in the pediatric age group in the meta-analysis, which may have confounded the results.

Our ranking of drug classes by the median proportion of PADRs caused is similar to that published by Kanjanarat et al. (2003) [94], who also identified cardiovascular drugs as the class most commonly associated with PADRs. We experienced similar difficulties to those reported by Kanjanarat et al. [94] in analysis in that a standard drug classification system was not used across the set of included studies, requiring amalgamation of some drug class data. However, our top 5 ranked drug classes were similar to those found in their previous work: cardiovascular drugs, analgesics, anticoagulants, opioids (combined with “analgesics” previously), and antibiotics/anti-infectives. “Psychoactive and central nervous system drugs” was the second-ranked drug class in the review by Kanjanarat et al. [94], and was a combination of sedatives, hypnotics, antidepressants, antipsychotic agents, benzodiazepines, and their combinations. We elected not to amalgamate these categories; however, this class likely would have been ranked high had we employed this approach. It is possible that bias in PADR reporting may influence the ranking of drugs, as our higher ranked classes of drugs are potentially associated with more severe AEs, have generally narrow therapeutic windows, and may be more likely to be administered for life-threatening or complex medical conditions in which PADR risk is higher.

There are limitations of the current review of note, several that are directly related to the design of the review, specifically that it was a systematic review of systematic reviews. We elected to conduct a review of reviews rather than a review of primary studies based on an initial scoping of the literature that indicated that there was an abundance of systematic reviews published in the area of the topic of interest. However, in our review of reviews, encountered considerable variability in approaches amongst the set of included reviews, as well as limitations in availability of detailed PADR data due to differences in the primary objectives of the included reviews. To capture the broadest range of PADR data, we had not excluded reviews based on their objective, resulting in considerable heterogeneity. To improve our ability to address the research questions of interest we modified our a priori approach and collected additional data directly from the primary studies originally reported by one or more of the 13 systematic reviews as having PADR data. As hoped, this strategy enriched the evidence available to inform responses to the review questions of interest. However, it should be noted that because we did not systematically review the primary study literature as a whole (i.e., our search strategy and screening methods were not targeted to identify primary studies) and included only primary studies that had previously been reported in systematic reviews with varying objectives, some primary studies relevant to our review objectives may have not been included. Because our included primary studies were potentially not comprehensive based on our review of reviews, the data reported herein may not be reflective of the entirety of the published literature. As well, because a systematic review of primary studies was not conducted, risk of bias assessments of primary studies were not conducted. The ROB in the primary studies likely varied; however, substantial heterogeneity in the myriad of other study-level factors, including study objectives and event detection methods, may have influenced PADR incidence estimates more than the ROB. This heterogeneity, as represented by measures of I^2^ commonly in excess of 90%, as well as potential missing primary studies indicate that estimates of PADR incidence derived from our analyses and subgroup analyses require cautious interpretation. As well, patient age categories reported in the primary studies were poorly reported or overlapped, adding further uncertainty to the findings from this subgroup meta-analysis. Our ability to synthesize the evidence for several of our secondary review questions was limited by non-standardized categorization within the primary literature of types of preventable events, medication process stages, PADR severity scores, and drug classes.

The initial motivation for this review was to estimate an overall incidence of in-hospital PADRs. Upon completion of our analyses, we recognized that given the substantial impact of various factors, including event detection method, patient age, setting, and PADR definitions and assessment tools used, a single overall PADR incidence estimate is not valid. A potentially more suitable approach for readers to consider would be to identify a primary study that most closely matches the reader’s intended context, with respect to setting and patient-level factors, and that also detects events using prospective methods, and to regard that study’s reported PADR incidence as potentially more accurate than an incidence synthesized from multiple heterogenous sources. In the Canadian context, no primary study has been published with the objective of estimating in-hospital PADR incidence using either prospective or retrospective methods. Amongst our included studies, King et al., 2003 [65] conducted an intervention study in pediatric inpatients in a Canadian hospital and measured PADR incidence pre- and post-intervention. However, voluntary reporting methods were used, which, although suitable for longitudinal comparative analyses, undoubtedly considerably underestimated the true incidence of PADRs. In 2002, the Canadian Adverse Events Study [2] (not included in our review) used retrospective chart review to estimate an incidence of in-hospital preventable adverse events in 20 Canadian hospitals; however, the measured events were caused both by errors related to healthcare management as well as drugs. Again, the method of event detection likely underestimated the true incidence, while inclusion of non-drug causes would have inflated the reported incidence above that of PADRs alone. In-hospital PADR incidence estimates published in non-Canadian studies may be more accurate.

## Conclusions

More well-conducted, well-reported primary studies that use accepted definitions and causality assessment tools need to be conducted in the Canadian context to address the lack of Canadian PADR data in the published literature. Globally, while several systematic reviews and a broad range of primary research studies exist that have sought to assess the occurrence of preventable adverse drug reactions (and related outcomes), there remains considerable diversity in methods and other factors that complicate the ability to establish a single overall estimate of PADR incidence. Rather than using an incidence estimate pooled from multiple heterogenous studies, readers may want to consider using incidence estimates reported in individual primary studies that incorporated prospective event detection methods and that were conducted in a similar context to the one of interest (i.e., country and hospital setting). Our findings from our secondary review questions and their general concordance with the existing literature suggest that interventions to reduce PADRs may be most effective when targeted at the use of specific drug classes in medical and ICU patients. However, substantial reductions in adverse events may be unlikely to occur without complex, multi-component interventions, including intensive institutional cultural change.

## Supporting information

S1 Dataset Data collected from the included systematic reviews. (XLSX)

S2 Dataset Data collected from the included primary studies. (XLSX)

S1 Fig Flow diagram of study selection process. (DOCX)

S1 Text Medline search strategy. (DOCX)

S2 Text Data extraction details. (DOCX)

S3 Text Details of the Jadad framework for discordant reviews. (DOCX)

S4 Text PRISMA checklist. (DOCX)

S5 Text AMSTAR-2 evaluations of included reviews. (DOCX)

S6 Text Additional analyses. (DOCX)

S7 Text Summary of stages of the medication process at which errors occurred that resulted in PADRs. (DOCX)

S8 Text Impact of system-level interventions on PADR incidence analyzed by vote count. (DOCX)

S9 Text Summary of severity of harm of the PADRs detected in primary studies. (DOCX)

S10 Text Included reviews and primary studies. (DOCX)

S11 Text Studies excluded during full-text screening. (DOCX)
