# Supplementary material for: Incidence, causes, and consequences of preventable adverse drug reactions occurring in inpatients: A systematic review of systematic reviews
Source: PLoS One. 2018 Oct 11;13(10):e0205426. doi: 10.1371/journal.pone.0205426 (PMC6181371; doi:10.1371/journal.pone.0205426)
Supplement: S1 Fig — (DOCX) [file pone.0205426.s003.docx]

**Appendix 4: Flow diagram of study selection process**

Potential primary studies reported in included reviews (n = 41)

Included primary studies

(n = 37)

Reviews included
(n = 13)

Full-text articles excluded, with reasons
(n = 232)

- Full text not available (n = 7)
- Setting not exclusive to inpatients/acute, ambulatory care, etc. (n = 43)
- Not exclusive to drug related adverse effects (n = 4)
- No report of preventable ADEs (n = 129)
- Not a systematic review (n = 49)

Records excluded
(n = 9,417)

Records screened after duplicates removed
(n = 9,662)

Full-text articles assessed for eligibility
(n = 245)

Additional records identified through other sources
(n = 3)

**Identification**

**Eligibility**

**Included**

**Screening**

Records identified through database searching
(n = 10,506)
