# Supplementary material for: Incidence, causes, and consequences of preventable adverse drug reactions occurring in inpatients: A systematic review of systematic reviews
Source: PLoS One. 2018 Oct 11;13(10):e0205426. doi: 10.1371/journal.pone.0205426 (PMC6181371; doi:10.1371/journal.pone.0205426)
Supplement: S1 Text — (DOCX) [file pone.0205426.s004.docx]

Appendix 1: Medline search strategy

Database: Epub Ahead of Print, In-Process & Other Non-Indexed Citations, Ovid MEDLINE(R) Daily and Ovid MEDLINE(R) <1946 to Present>

Search Strategy:

--------------------------------------------------------------------------------

1 (PADR or PADRs or PADE or PADEs).tw,kw. (316)

2 exp Drug Therapy/ae (59504)

3 limit 2 to yr="1966-1991" (10067)

4 ((drug or drugs or drug-related or medication? or pharmaceutical* or prescription*) adj3 ((monitor* or surveillance) adj1 (postmarket* or post-market*))).tw,kw. (183)

5 ((pharmacovigilan* or pharmaco-vigilan* or pharmcosurveillance or pharmaco-surveillance) and (adverse* or complication* or harm or harms or harmful or harmfull or harming or injurious or side effect* or undesirable or teratogen* or toxic* or interaction? or reaction?)).tw,kw. (2185)

6 Adverse Drug Reaction Reporting Systems/ (6184)

7 exp "Drug-Related Side Effects and Adverse Reactions"/ (97745)

8 (ADR or ADRs or ADE or ADEs).tw,kw. (9884)

9 ((drug or drugs or drug-related or medication? or pharmaceutical* or prescription*) adj3 (adverse* or complication* or harm or harms or harmful or harmfull or harming or injurious or side effect* or undesirable or teratogen* or toxic* or interaction? or reaction?)).tw,kw. (92957)

10 exp Medication Errors/ (12877)

11 ((dispens* or dosag* or drug or drugs or drug-related or medication? or pharmaceutical* or prescrib* or prescription*) adj3 (discrepanc* or error* or mistake* or omission* or overdos* or oversight? or wrong*)).tw,kw. (9994)

12 Prescription Drugs/ae, po (874)

13 exp Nonprescription Drugs/ae, po (1336)

14 ci.fs. (520384)

15 or/3-14 (696130)

16 exp "Drug-Related Side Effects and Adverse Reactions"/pc (5453)

17 exp Medication Errors/pc (5524)

18 preventab*.tw,kw. (21577)

19 avoidab*.tw,kw. (6702)

20 stoppab*.tw,kw. (6)

21 ((avoid* or control* or prevent* or stop*) adj5 (discrepanc* or error* or mistake* or omission* or overdos* or oversight? or wrong*)).tw,kw. (14876)

22 ((avoid* or control* or prevent* or stop*) adj5 (ADR or ADRs or ADE or ADEs or adverse* or complication* or harm or harms or harmful or harmfull or harming or injurious or side effect* or undesirable or teratogen* or toxic* or interaction? or reaction? or unsafe*)).tw,kw. (126226)

23 or/16-22 (173239)

24 15 and 23 (24449)

25 1 or 24 (24734)

26 Adverse Drug Reaction Reporting Systems/sn (1139)

27 exp "Drug-Related Side Effects and Adverse Reactions"/ep (5120)

28 exp Medication Errors/sn (2594)

29 Prevalence/ (220476)

30 Incidence/ (206220)

31 (frequen* or incidenc* or occurrence* or prevalent* or prevalence*).tw,kw. (2470205)

32 or/26-31 (2584868)

33 15 and 32 (111340)

34 25 or 33 (129168)

35 exp Animals/ not (exp Animals/ and Humans/) (4247972)

36 34 not 35 (104708)

37 (comment or editorial or interview or news or newspaper article).pt. (1151809)

38 (letter not (letter and randomized controlled trial)).pt. (917616)

39 36 not (37 or 38) (101095)

40 limit 39 to systematic reviews (4295)

41 meta analysis.pt. (66122)

42 exp meta-analysis as topic/ (14910)

43 (meta-analy* or metanaly* or metaanaly* or met analy* or integrative research or integrative review* or integrative overview* or research integration or research overview* or collaborative review*).tw,kw. (98096)

44 (systematic review* or systematic overview* or evidence-based review* or evidence-based overview* or (evidence adj3 (review* or overview*)) or meta-review* or meta-overview* or meta-synthes* or "review of reviews" or technology assessment* or HTA or HTAs).tw,kw. (119761)

45 exp Technology assessment, biomedical/ (9697)

46 (cochrane or health technology assessment or evidence report).jw. (14486)

47 (network adj (MA or MAs)).tw,kw. (2)

48 (NMA or NMAs).tw,kw. (1396)

49 indirect comparison?.tw,kw. (1072)

50 (indirect treatment* adj1 comparison?).tw,kw. (99)

51 (mixed treatment* adj1 comparison?).tw,kw. (323)

52 (multiple treatment* adj1 comparison?).tw,kw. (67)

53 (multi-treatment* adj1 comparison?).tw,kw. (0)

54 simultaneous comparison?.tw,kw. (382)

55 mixed comparison?.tw,kw. (11)

56 or/41-55 (218326)

57 39 and 56 (3709)

58 40 or 57 (5114)

59 limit 58 to (english or french) (4868)
