# Supplementary material for: Incidence, causes, and consequences of preventable adverse drug reactions occurring in inpatients: A systematic review of systematic reviews
Source: PLoS One. 2018 Oct 11;13(10):e0205426. doi: 10.1371/journal.pone.0205426 (PMC6181371; doi:10.1371/journal.pone.0205426)
Supplement: S2 Text — (DOCX) [file pone.0205426.s005.docx]

Appendix 2: Data extraction details

**Data types extracted from the included systematic reviews and primary studies**

| **Systematic reviews** | **Primary studies** |
| --- | --- |
| Manuscript data (authors, year of publication)  Objective  Was a search strategy reported?  Number of databases searched  Language, geographic, and date restrictions  Inclusion/exclusion criteria  Intervention(s) of interest (if applicable)  Patient population of interest  Main outcomes of interest  Number of included studies (total)  Number of studies/RCTs/non-randomized studies reporting PADR data  Funding type/source detail  Individual PADR primary study details and reported PADR incidence  Pooled PADR estimate | Manuscript data (authors, year of publication, country of conduct)  Study design  Setting (hospital type, number of beds, within hospital setting (i.e., wards, ICUs, etc.), clinical discipline)  Patient age, disease type  Medication class/type  Event detection method  Event type detected (i.e., MEs, ADRs/ADEs, PADRs/PADEs)  ADR definition  Causality assessment tool  Preventability assessment tool  Intervention(s) evaluated (if applicable)  PADR cause(s), severity  PADR incidence reported (all units of measure) |
