# Supplementary material for: Incidence, causes, and consequences of preventable adverse drug reactions occurring in inpatients: A systematic review of systematic reviews
Source: PLoS One. 2018 Oct 11;13(10):e0205426. doi: 10.1371/journal.pone.0205426 (PMC6181371; doi:10.1371/journal.pone.0205426)
Supplement: S3 Text — (DOCX) [file pone.0205426.s006.docx]

Appendix 3: Details of the Jadad framework for discordant reviews

| **Domain** | **Element** |
| --- | --- |
| Clinical question | Populations of patients |
|  | Interventions |
|  | Outcome measures |
|  | Settings |
| Study selection and inclusion | Selection criteria |
|  | Application of the selection criteria |
|  | Strategies to search literature |
| Data extraction | Methods to measure outcomes |
|  | End points |
|  | Human error (random or systematic) |
| Assessment of study quality | Methods to assess quality |
|  | Interpretations of quality assessment |
|  | Methods to incorporate quality assessments in review |
| Assessment of the ability to combine studies | Statistical methods |
|  | Clinical criteria to judge the ability to combine studies |
| Statistical methods for data synthesis |  |
