# Supplementary material for: Incidence, causes, and consequences of preventable adverse drug reactions occurring in inpatients: A systematic review of systematic reviews
Source: PLoS One. 2018 Oct 11;13(10):e0205426. doi: 10.1371/journal.pone.0205426 (PMC6181371; doi:10.1371/journal.pone.0205426)
Supplement: S5 Text — (DOCX) [file pone.0205426.s008.docx]

**Appendix 5: AMSTAR-2 Evaluations of Included Reviews**

**Assessment of methodological quality/risk of bias in the included systematic reviews using the AMSTAR 2 tool**

| Review | AMSTAR 2 element | | | | | | | | | | | | | | | | | |
| --- | --- | --- | --- | --- | --- | --- | --- | --- | --- | --- | --- | --- | --- | --- | --- | --- | --- | --- |
|  | 1 | 2 | 3 | 4 | 5 | 6 | 7 | 8 | 9a | 9b | 10 | 11a | 11b | 12 | 13 | 14 | 15 | 16 |
| Kanagaratnam 2016 [13] |  |  |  |  |  |  |  |  | — |  |  | — | — | — |  |  |  |  |
| Boeker 2015 [16]* |  |  |  |  |  | — |  |  | — |  |  | — |  |  |  |  |  |  |
| Maaskant 2015 [18] |  |  |  |  |  |  |  |  |  |  |  | — | — |  |  |  |  |  |
| Salmasi 2015 [20] |  |  |  |  |  |  |  |  | — |  |  | — | — | — |  |  |  |  |
| Wang 2015 [22] |  |  |  |  |  |  |  |  | — |  |  | — |  |  |  |  |  |  |
| Acheampong 2014 [25] |  |  |  |  |  |  |  |  | — |  |  | — | — | — |  |  |  |  |
| Manias 2014 [26] |  |  |  |  |  |  |  |  | — |  |  | — |  |  |  |  |  |  |
| Nuckols 2014 [28] |  |  |  |  |  |  |  |  | — |  |  | — |  |  |  |  |  |  |
| Boeker 2013 [29] |  |  |  |  |  |  |  |  | — |  |  | — | — | — |  |  |  |  |
| Hakkarainen 2012 [30] |  |  |  |  |  |  |  |  | — |  |  | — |  |  |  |  |  |  |
| Manias 2012 [35] |  |  |  |  |  |  |  |  |  |  |  | — | — | — |  |  |  |  |
| Damiani 2009 [37] |  |  |  |  |  |  |  |  | — |  |  | — | — | — |  |  |  |  |
| Hodgkinson 2006 [38] |  |  |  |  |  |  |  |  |  |  |  | — | — | — |  |  |  |  |

Green = Yes; Yellow = Partial yes; Red = No; White = Not applicable (e.g., no RCTs included, no non-randomized studies included, a meta-analysis was not conducted)

*Boeker 2015: meta-analysis of individual patient data

**AMSTAR 2 elements:**

1. Did the research questions and inclusion criteria for the review include the components of PICO?
2. Did the report of the review contain an explicit statement that the review methods were established prior to the conduct of the review?
3. Did the review authors explain their selection of the study designs for inclusion in the review?
4. Did the review authors use a comprehensive literature search strategy?
5. Did the review authors perform study selection in duplicate?
6. Did the review authors perform data extraction in duplicate?
7. Did the review authors provide a list of excluded studies and justify the exclusions?
8. Did the review authors describe the included studies in adequate detail?
   1. Did the review authors use a satisfactory technique for assessing the risk of bias (RoB) in individual studies that were included in the review (RCTs)?
   2. Did the review authors use a satisfactory technique for assessing the risk of bias (RoB) in individual studies that were included in the review (NRS)?
9. Did the review authors report on the sources of funding for the included studies?
   1. If meta-analysis of RCTs was performed did the authors use appropriate methods for statistical combination of results?
   2. If meta-analysis of NRSIs was performed did the authors use appropriate methods for statistical combination of results?
10. If meta-analysis was performed, did the review authors assess the potential impact of RoB in individual studies on the results of the meta-analysis or other evidence synthesis?
11. Did the review authors account for RoB in individual studies when interpreting/discussing the results of the review?
12. Did the review authors provide a satisfactory explanation for, and discussion of, any heterogeneity observed in the results of the review?
13. Did the review authors carry out an adequate investigation of publication bias, and discuss its likely impact on the results of the review?
14. Did the review authors report any potential sources of conflict of interest, including any funding they received for conducting the review?
