# Supplementary material for: Incidence, causes, and consequences of preventable adverse drug reactions occurring in inpatients: A systematic review of systematic reviews
Source: PLoS One. 2018 Oct 11;13(10):e0205426. doi: 10.1371/journal.pone.0205426 (PMC6181371; doi:10.1371/journal.pone.0205426)
Supplement: S6 Text — (DOCX) [file pone.0205426.s009.docx]

**Appendix 6: Additional analyses**

**Subgroup meta-analysis by event type of interest (PADRs per 100 patients)**

**Figure**: Forest plot of subgroup analysis by the event type of interest in the included primary study

**Subgroup meta-analysis by ADR/ADE definition (PADRs per 100 patients)**

**Figure**: Forest plot of subgroup analysis by the ADR or ADE definition used in the included primary study

References: Edwards and Aronson [2]; NCC MERP [3]; WHO [1]

**Subgroup meta-analysis by causality assessment tool (PADRs per 100 patients)**

**Figure**: Forest plot of subgroup analysis by the causality assessment tool used in the included primary study

References: Brigham and Women’s Hospital [41,42,48], Karch and Lasagna [63], Naranjo tool [64]

**Subgroup meta-analysis by preventability assessment tool (PADRs per 100 patients)**

**Figure**: Forest plot of subgroup analysis by the preventability assessment tool used in the included primary study

References: Hallas [11], Schumock and Thornton [9]

**Additional data and analyses: PADR Incidence per 1,000 patient-days**

**Figure**: Distribution of data in 16 primary studies that reported PADRs per 1,000 patient-days^a^ [19,24,36,41–43,46,49,52,53,59,61,65,67–69]

^a^ The extreme outlier [67] included events resulting in increased patient monitoring but no harm as well as events that caused patient harm

**Figure**: Pooled PADR incidence per 1,000 patient-days (outlier removed)

When the outlier was retained in the dataset, the pooled PADR incidence changed very little: 3.80 (2.81–4.79) PADRs per 1,000 patient-days.
