# Supplementary material for: Incidence, causes, and consequences of preventable adverse drug reactions occurring in inpatients: A systematic review of systematic reviews
Source: PLoS One. 2018 Oct 11;13(10):e0205426. doi: 10.1371/journal.pone.0205426 (PMC6181371; doi:10.1371/journal.pone.0205426)
Supplement: S7 Text — (DOCX) [file pone.0205426.s010.docx]

**Appendix 7: Summary of stages of the medication process at which errors occurred that resulted in PADRs**

| Primary study | Ordering | Transcription | Dispensing | Drug preparation, handling, etc. | Administration | Contraindication (known allergy) | Omission of dose/ medication | Wrong medication | Wrong dose | Inappropriate medication | Insufficient monitoring | Wrong timing | Unnecessary medication |
| --- | --- | --- | --- | --- | --- | --- | --- | --- | --- | --- | --- | --- | --- |
| Aljadhey 2013 | 96% |  | 4% |  |  |  |  |  |  |  |  |  |  |
| de Boer 2013 | 88%^a^ |  |  |  |  |  |  |  |  |  |  |  |  |
| Menendez 2012 |  |  |  |  |  |  | 48%^a^ |  | 24%^a^ |  |  |  |  |
| Morimoto 2011 | 34% |  |  |  | 11% |  |  |  |  |  | 55% |  |  |
| Chapuis 2010 |  |  |  |  |  |  |  | 29% (wrong dose, medication, form, etc.)^a^ | | |  |  |  |
| Klopotowska 2010 |  |  |  |  |  |  | 50% “drug or omission error” | |  |  | 50% |  |  |
| Berga Cullere 2009 |  |  |  | 5%^b^ | 1% ^b^ |  | 36% ^b^ | 17% ^b^ | 29% ^b^ | 14% ^b^ | 7% ^b^ | 3% ^b^ | 2% ^b^ |
| Morriss 2009 | 8% ^a^ |  |  |  | 3% ^a^ |  | 36% ^a^ |  | 13% ^a^ |  |  | 31% ^a^ |  |
| Van Doormal 2009 |  | 13% |  |  |  |  | 54% “therapeutic errors” | | 33% |  |  |  |  |
| Handler 2008 | 69% “prescribing stage” ^a^ | | |  |  |  |  |  |  |  |  |  |  |
| Nuckols 2008 | 51% |  |  |  | 14% |  |  |  |  |  | 35% |  |  |
| Colpaert 2006 |  |  |  |  |  |  |  |  | “mainly dosing errors” ^a^ |  |  |  |  |
| Hintong 2005 |  |  |  |  |  |  |  | 57% | 43% |  |  |  |  |
| Sangtawesin 2003 |  |  |  |  |  |  |  |  | 100% |  |  |  |  |
| Mullett 2001 |  |  |  |  |  | 100% |  |  |  |  |  |  |  |
| Bates 1995 | 56% | 6% | 3% |  | 34% |  |  |  |  |  |  |  |  |
| Bates 1993 |  |  |  |  |  | 7% ^a^ |  |  |  |  |  |  |  |

^a^ Reported data totalled <100%

^b^ Reported data totalled >100%
