# Supplementary material for: Incidence, causes, and consequences of preventable adverse drug reactions occurring in inpatients: A systematic review of systematic reviews
Source: PLoS One. 2018 Oct 11;13(10):e0205426. doi: 10.1371/journal.pone.0205426 (PMC6181371; doi:10.1371/journal.pone.0205426)
Supplement: S8 Text — (DOCX) [file pone.0205426.s011.docx]

**Appendix 8: Impact of system-level interventions on PADR incidence analyzed by vote count**

| **Intervention (number of studies) [citations]** | **Effect of intervention on PADR incidence** | | |
| --- | --- | --- | --- |
|  | **Number of studies demonstrating significant decrease in PADR incidence** | **Number of studies demonstrating no significant change in PADR incidence** | **Number of studies demonstrating decrease in PADR incidence, but significance was not reported** |
| Computerized physician order entry (n = 10) [48,47,50,43,51,58,67,19,69,49] | 3 | 5 | 2 |
| Clinical decision support systems (n = 2) [45,65] | 0 | 2 | 0 |
| Pharmacist participation (n = 3) [24,36,61] | 2 | 1 | 0 |
| Automation of drug dispensing or administration (n = 3) [46,59,60] | 1 | 2 | 0 |
| Cultural-change interventions (n = 2) [27,68] | 2 | 0 | 0 |

**Studies evaluating computerized physician order entry (CPOE)**

1. Leung et al. (2012) [48] evaluated a CPOE in community hospitals. The PADR incidence decreased at every site, from a weighted average of 10.6 to 7.0 per 100 patients (p = 0.007).
2. Menendez et al. (2012) [47] evaluated a CPOE in an unknown setting. PADRs were reduced to 10% of the pre-intervention period (Relative Risk = 0.10; 99% CI: 0.20–0.05).
3. Van Doormaal et al. (2009) [50] evaluated a CPOE with clinical decision support in general medicine wards. The proportion of patients suffering at least one PADE decreased from 15.5% to 7.3% post-intervention (not significant).
4. Walsh et al. (2008) [43] evaluated a CPOE in pediatrics. The rate of PADEs did not change significantly (Incidence Rate Ratio = 0.83; 95% CI: 0.37–1.87).
5. Weant et al. (2007) [51] evaluated a CPOE in a neurosurgical ICU. PADR incidence decreased from 0.137 to 0.0152 per 1,000 doses post-intervention (significance not reported).
6. Bradley et al. (2006) [58] evaluated a CPOE in medical-surgical and intensive care units. PADRs decreased from 0.41 to 0.07 per 100 patients (significance not reported).
7. Colpaert et al. (2006) [67] evaluated a CPOE in an ICU. Total ADEs, which included some that did not cause harm, decreased to 15% of pre-intervention levels (p < 0.01).
8. King et al. (2003) [19] evaluated a CPOE in pediatric wards. Implementation had no effect on the rate of PADRs (Incidence Rate Ratio = 1.30; 95% CI: 0.47–3.52).
9. Bates et al. (1999) [69] evaluated a CPOE with decision support features that was serially improved over the study period and compared to a paper-based system. PADR rate decreased from 2.9 in the paper-based system to 1.1 PADRs/1,000 patient-days in the final study period, but the significance of the decreasing trend was equivocal (p = 0.05).
10. Bates et al. (1998) [49] evaluated a CPOE and a combination of CPOE + “team intervention” (changing the role of the pharmacist, standardization of several processes, and a pharmacy communication log) against a baseline of no intervention. They found that PADEs decreased 17%, but the difference was not statistically significant (p = 0.37).

**Studies evaluating clinical decision support systems**

1. Gurwitz et al. (2008) [65] evaluated a CPOE with or without a computerized clinical decision support system in a LTC setting. The baseline setting already had a CPOE. The implementation of the computerized clinical decision support system did not influence PADR incidence (Relative Risk = 1.01; 95% CI: 0.81–1.30).
2. Mullett et al. (2001) [45] evaluated a computerized anti-infective decision support program in a PICU. Only 1 PADR was found in each study period (in 487 and 631 patients, respectively).

**Studies evaluating pharmacist participation**

1. Klopotowska et al. (2010) [36] evaluated the on-ward participation of hospital pharmacists in an ICU. PADR incidence decreased from 4.0 to 1.0 per 1,000 patient-days; however, this difference was not statistically significant (p = 0.25).
2. Kucukarslan et al. (2003) [61] evaluated pharmacist participation on rounding teams in general medicine units. The rate of PADEs was reduced to 22% of the pre-intervention rate (from 26.5 to 5.7 per 1,000 patient-days) (p = 0.02).
3. Leape et al. (1999) [24] evaluated pharmacist participation on medical rounds in the ICU. The rate of PADRs decreased from 10.4 pre-intervention to 3.5 PADRs per 1,000 patient-days post-intervention, with a control unit remaining unchanged at 10.9 and 12.5 PADRs per 1,000 patient-days pre- and post-intervention. (p < 0.001)

**Studies evaluating automation of drug administration/dispensing**

1. Chapuis et al. (2010) [60] evaluated an automated drug dispensing system in an ICU. The intervention reduced MEs not causing harm by 35%; however, MEs causing harm (PADRs) were unaffected.
2. Morriss et al. (2009) [46] evaluated a barcode medication administration system in a NICU. The PADR incidence was halved from 0.86 to 0.43 PADRs per 1,000 doses (p = 0.008).
3. Nuckols et al. (2008) [59] evaluated programmable infusion pumps in ICUs. No significant difference was found in the rate of preventable IV-ADEs between groups (adjusted mean difference = 0.04 per 1,000 patient-days; p = 0.96).

**Studies evaluating cultural-change interventions**

1. Abstoss et al. (2011) [27] evaluated 7 overlapping interventions to improve medication safety and reporting, including 4 to induce institutional cultural change (e.g., poster tracking ‘days since last ME resulting in harm,’ continuous slideshow of performance metrics, didactic curricula, emails summarizing MEs) and 3 system-level interventions (e.g., a CPOE, unit-based pharmacy technicians for medication delivery, patient safety report form streamlining). They found that subsequent to implementation, the ME reporting rate increased 25% and the rate of PADRs decreased significantly by 71% (p < 0.01).
2. Cohen et al. (2005) [68] evaluated a medication safety program in a community hospital, which included intensive work on cultural change to increase ME reporting and the introduction of a number of drug protocols and standardized procedures. As a result, the relative risk for ADEs associated with harm was reduced to 12% of pre-intervention levels (p < 0.001).
