# Supplementary material for: Incidence, causes, and consequences of preventable adverse drug reactions occurring in inpatients: A systematic review of systematic reviews
Source: PLoS One. 2018 Oct 11;13(10):e0205426. doi: 10.1371/journal.pone.0205426 (PMC6181371; doi:10.1371/journal.pone.0205426)
Supplement: S9 Text — (DOCX) [file pone.0205426.s012.docx]

**Appendix 9: Summary of severity of harm of the PADRs detected in primary studies**

| Primary study | NCC MERP categories [3] | | | | | Folli 1987 [70] | | | | CTCAE scale [71] | | | | Bennett 1977 [72] | | | Custom definitions | | | | | | | | | |
| --- | --- | --- | --- | --- | --- | --- | --- | --- | --- | --- | --- | --- | --- | --- | --- | --- | --- | --- | --- | --- | --- | --- | --- | --- | --- | --- |
|  | E | F | G | H | I | Signif | Ser | Life-threat | Ftl | Mild | Mod | Sev | Life-threat | Mild | Mod | Sev | Less sev | More sev | Non-ser | Ser | Trans | Life-threat | Signif | Ser | Life-threat | Ftl |
| Abstoss 2011 [27] | 8% of MEs | 0% of MEs | 0% of MEs | 0% of MEs | 0% of MEs |  |  |  |  |  |  |  |  |  |  |  |  |  |  |  |  |  |  |  |  |  |
| Chapuis 2010 [60] | 49% of MEs | 37% of MEs | 0% of MEs | 14% of MEs | — |  |  |  |  |  |  |  |  |  |  |  |  |  |  |  |  |  |  |  |  |  |
| Berga Cullere 2009 [39] | 85% | 11% | 0% | 4% | 0% |  |  |  |  |  |  |  |  |  |  |  |  |  |  |  |  |  |  |  |  |  |
| Van Doormaal 2009 [50] | 89% | 8% | 2% | 1% | — |  |  |  |  |  |  |  |  |  |  |  |  |  |  |  |  |  |  |  |  |  |
| Bates 1999 [69] |  |  |  |  |  | 0% | 80% | 20% | 0% |  |  |  |  |  |  |  |  |  |  |  |  |  |  |  |  |  |
| Bates 1998 [49] |  |  |  |  |  | 64% | 22% | 15% | 0% |  |  |  |  |  |  |  |  |  |  |  |  |  |  |  |  |  |
| Bates 1995 [52] |  |  |  |  |  | 37% | 43% | 20% | 0% |  |  |  |  |  |  |  |  |  |  |  |  |  |  |  |  |  |
| de Boer 2013 [40] |  |  |  |  |  |  |  |  | 0% | 46% | 29% | 17% | 8% |  |  |  |  |  |  |  |  |  |  |  |  |  |
| Klopotowska 2010 [36] | 100% were E-F | |  |  |  |  |  |  |  | 0% | 0% | 2 of 2 | 0% |  |  |  |  |  |  |  |  |  |  |  |  |  |
| Leung 2012 [48] |  |  |  |  |  |  |  |  |  |  |  |  |  |  |  |  |  |  |  |  |  |  | 25% | 57% | 17% | 1% |
| Gurwitz 2008 [65] |  |  |  |  |  |  |  |  |  |  |  |  |  |  |  |  | 54% | 46% |  |  |  |  |  |  |  |  |
| Handler 2008 [66] |  |  |  |  |  |  |  |  |  |  |  |  |  |  |  |  |  |  | 81% | 19% |  |  |  |  |  |  |
| Hintong 2005 [21] |  |  |  |  |  |  |  |  |  |  |  |  |  |  |  |  |  |  |  |  | 93% | 7% |  |  |  |  |
| Pearson 1994 [34] |  |  |  |  |  |  |  |  |  |  |  |  |  | 0% | 68% | 32% |  |  |  |  |  |  |  |  |  |  |

“Ftl” = fatal, “Life-threat” = life-threatening, “Mod” = moderate, “Ser” = serious, “Sev” = severe, “Signif” = significant, “Trans” = transient
