# Supplementary material for: Incidence, causes, and consequences of preventable adverse drug reactions occurring in inpatients: A systematic review of systematic reviews
Source: PLoS One. 2018 Oct 11;13(10):e0205426. doi: 10.1371/journal.pone.0205426 (PMC6181371; doi:10.1371/journal.pone.0205426)
Supplement: S10 Text — (DOCX) [file pone.0205426.s013.docx]

**Appendix 10: Included reviews and primary studies**

***Included Systematic Reviews***

1. Acheampong, F., Anto, B. P. & Koffuor, G. A. Medication safety strategies in hospitals–A systematic review. *International Journal of Risk & Safety in Medicine* **26,** 117–131 (2014).

2. Boeker, E. B. *et al.* Occurrence and preventability of adverse drug events in surgical patients: a systematic review of literature. *BMC health services research* **13,** 1 (2013).

3. Boeker, E. B. *et al.* An individual patient data meta-analysis on factors associated with adverse drug events in surgical and non-surgical inpatients: An IPDMA on factors associated with ADEs in surgical and non-surgical inpatients. *British Journal of Clinical Pharmacology* **79,** 548–557 (2015).

4. Damiani, G., Pinnarelli, L., Scopelliti, L., Sommella, L. & Ricciardi, W. A review on the impact of systematic safety processes for the control of error in medicine. *Medical Science Monitor* **15,** RA157–RA166 (2009).

5. Hakkarainen, K. M., Hedna, K., Petzold, M. & Hägg, S. Percentage of Patients with Preventable Adverse Drug Reactions and Preventability of Adverse Drug Reactions – A Meta-Analysis. *PLoS ONE* **7,** e33236 (2012).

6. Hodgkinson, B., Koch, S., Nay, R. & Nichols, K. Strategies to reduce medication errors with reference to older adults. *International Journal of Evidence-Based Healthcare* **4,** 2–41 (2006).

7. Kanagaratnam, L. *et al.* Adverse drug reactions in elderly patients with cognitive disorders: A systematic review. *Maturitas* **85,** 56–63 (2016).

8. Maaskant, J. M. *et al.* Interventions for reducing medication errors in children in hospital. in *Cochrane Database of Systematic Reviews* (ed. The Cochrane Collaboration) (John Wiley & Sons, Ltd, 2015).

9. Manias, E., Kinney, S., Cranswick, N., Williams, A. & Borrott, N. Interventions to reduce medication errors in pediatric intensive care. *Annals of Pharmacotherapy* **48,** 1313–1331 (2014).

10. Manias, E., Williams, A. & Liew, D. Interventions to reduce medication errors in adult intensive care: a systematic review: Interventions to reduce medication errors. *British Journal of Clinical Pharmacology* **74,** 411–423 (2012).

11. Nuckols, T. K. *et al.* The effectiveness of computerized order entry at reducing preventable adverse drug events and medication errors in hospital settings: a systematic review and meta-analysis. *Syst Rev* **3,** 56 (2014).

12. Salmasi, S., Khan, T. M., Hong, Y. H., Ming, L. C. & Wong, T. W. Medication Errors in the Southeast Asian Countries: A Systematic Review. *PLOS ONE* **10,** e0136545 (2015).

13. Wang, T. *et al.* Effect of critical care pharmacist’s intervention on medication errors: A systematic review and meta-analysis of observational studies. *Journal of Critical Care* **30,** 1101–1106 (2015).

***Included Primary Studies***

1. Abstoss, K. M. *et al.* Increasing medication error reporting rates while reducing harm through simultaneous cultural and system-level interventions in an intensive care unit. *BMJ Quality & Safety* **20,** 914–922 (2011).

2. Aljadhey, H. *et al.* Incidence of adverse drug events in an academic hospital: a prospective cohort study. *International Journal for Quality in Health Care* **25,** 648–655 (2013).

3. Baniasadi, S., Fahimi, F. & Shalviri, G. Developing an adverse drug reaction reporting system at a teaching hospital. *Basic Clin Pharmacol Toxicol* **102,** 408–411 (2008).

4. Bates, D. W. *et al.* Incidence of adverse drug events and potential adverse drug events: implications for prevention. *Jama* **274,** 29–34 (1995).

5. Bates, D. W. *et al.* Effect of computerized physician order entry and a team intervention on prevention of serious medication errors. *JAMA* **280,** 1311–1316 (1998).

6. Bates, D. W., Leape, L. L. & Petrycki, S. Incidence and preventability of adverse drug events in hospitalized adults. *J Gen Intern Med* **8,** 289–294 (1993).

7. Bates, D. W. *et al.* The impact of computerized physician order entry on medication error prevention. *J Am Med Inform Assoc* **6,** 313–321 (1999).

8. Berga Culleré, C. *et al.* [Detecting adverse drug events during the hospital stay]. *Farm Hosp* **33,** 312–323 (2009).

9. Bradley, V. M., Steltenkamp, C. L. & Hite, K. B. Evaluation of reported medication errors before and after implementation of computerized practitioner order entry. *J Healthc Inf Manag* **20,** 46–53 (2006).

10. Chapuis, C. *et al.* Automated drug dispensing system reduces medication errors in an intensive care setting: *Critical Care Medicine* **38,** 2275–2281 (2010).

11. Cohen, M. M. Medication safety program reduces adverse drug events in a community hospital. *Quality and Safety in Health Care* **14,** 169–174 (2005).

12. Colpaert, K. *et al.* Impact of computerized physician order entry on medication prescription errors in the intensive care unit: a controlled cross-sectional trial. *Critical Care* **10,** 1 (2006).

13. Davies, E. C., Green, C. F., Mottram, D. R. & Pirmohamed, M. Adverse drug reactions in hospital in-patients: a pilot study. *J Clin Pharm Ther* **31,** 335–341 (2006).

14. Davies, E. C. *et al.* Adverse drug reactions in hospital in-patients: a prospective analysis of 3695 patient-episodes. *PLoS One* **4,** e4439 (2009).

15. de Boer, M. *et al.* Adverse drug events in surgical patients: an observational multicentre study. *Int J Clin Pharm* **35,** 744–752 (2013).

16. Dormann, H. *et al.* Readmissions and adverse drug reactions in internal medicine: the economic impact. *J Intern Med* **255,** 653–663 (2004).

17. Gholami, K. & Shalviri, G. Factors associated with preventability, predictability, and severity of adverse drug reactions. *Ann Pharmacother* **33,** 236–240 (1999).

18. Gurwitz, J. H. *et al.* Effect of computerized provider order entry with clinical decision support on adverse drug events in the long-term care setting. *J Am Geriatr Soc* **56,** 2225–2233 (2008).

19. Handler, S. M. *et al.* Assessing the performance characteristics of signals used by a clinical event monitor to detect adverse drug reactions in the nursing home. in *AMIA* (2008).

20. Hintong, T., Chau-In, W., Thienthong, S. & Nakcharoenwaree, S. An analysis of the drug error problem in the Thai Anesthesia Incidents Study (THAI Study). *J Med Assoc Thai* **88 Suppl 7,** S118-127 (2005).

21. King, W. J., Paice, N., Rangrej, J., Forestell, G. J. & Swartz, R. The effect of computerized physician order entry on medication errors and adverse drug events in pediatric inpatients. *Pediatrics* **112,** 506–509 (2003).

22. Klopotowska, J. E. *et al.* On-ward participation of a hospital pharmacist in a Dutch intensive care unit reduces prescribing errors and related patient harm: an intervention study. *Crit Care* **14,** R174 (2010).

23. Kucukarslan, S. N., Peters, M., Mlynarek, M. & Nafziger, D. A. Pharmacists on rounding teams reduce preventable adverse drug events in hospital general medicine units. *Arch. Intern. Med.* **163,** 2014–2018 (2003).

24. Laroche, M.-L. *et al.* Adverse drug reactions in patients with Alzheimer’s disease and related dementia in France: a national multicentre cross-sectional study: ADVERSE DRUG REACTIONS IN DEMENTIA. *Pharmacoepidemiology and Drug Safety* n/a-n/a (2013). doi:10.1002/pds.3471

25. Leape, L. L. *et al.* Pharmacist participation on physician rounds and adverse drug events in the intensive care unit. *Jama* **282,** 267–270 (1999).

26. Leung, A. A. *et al.* Impact of vendor computerized physician order entry in community hospitals. *J Gen Intern Med* **27,** 801–807 (2012).

27. Menendez, M. D. *et al.* Impact of computerized physician order entry on medication errors. *Rev Calid.Asist.* **27,** 334–340 (2012).

28. Morimoto, T. *et al.* Incidence of Adverse Drug Events and Medication Errors in Japan: the JADE Study. *Journal of General Internal Medicine* **26,** 148–153 (2011).

29. Morriss, F. H. *et al.* Effectiveness of a Barcode Medication Administration System in Reducing Preventable Adverse Drug Events in a Neonatal Intensive Care Unit: A Prospective Cohort Study. *The Journal of Pediatrics* **154,** 363–368.e1 (2009).

30. Mullett, C. J., Evans, R. S., Christenson, J. C. & Dean, J. M. Development and impact of a computerized pediatric antiinfective decision support program. *Pediatrics* **108,** e75–e75 (2001).

31. Nuckols, T. K. *et al.* Programmable Infusion Pumps in ICUs: An Analysis of Corresponding Adverse Drug Events. *Journal of General Internal Medicine* **23,** 41–45 (2008).

32. Pearson, T. F. *et al.* Factors associated with preventable adverse drug reactions. *Am J Hosp Pharm* **51,** 2268–2272 (1994).

33. Pourseyed, S. *et al.* Adverse drug reactions in patients in an Iranian department of internal medicine. *Pharmacoepidemiol Drug Saf* **18,** 104–110 (2009).

34. Sangtawesin, V., Kanjanapattanakul, W., Srisan, P., Nawasiri, W. & Ingchareonsunthorn, P. Medication errors at Queen Sirikit National Institute of Child Health. *Journal of the Medical Association of Thailand= Chotmaihet thangphaet* **86,** S570–5 (2003).

35. van Doormaal, J. E. *et al.* The influence that electronic prescribing has on medication errors and preventable adverse drug events: an interrupted time-series study. *J Am Med Inform Assoc* **16,** 816–825 (2009).

36. Walsh, K. E. *et al.* Effect of Computer Order Entry on Prevention of Serious Medication Errors in Hospitalized Children. *PEDIATRICS* **121,** e421–e427 (2008).

37. Weant, K. A., Cook, A. M. & Armitstead, J. A. Medication-error reporting and pharmacy resident experience during implementation of computerized prescriber order entry. *Am J Health Syst Pharm* **64,** 526–530 (2007).
