# Supplementary material for: Incidence, causes, and consequences of preventable adverse drug reactions occurring in inpatients: A systematic review of systematic reviews
Source: PLoS One. 2018 Oct 11;13(10):e0205426. doi: 10.1371/journal.pone.0205426 (PMC6181371; doi:10.1371/journal.pone.0205426)
Supplement: S11 Text — (DOCX) [file pone.0205426.s014.docx]

**Appendix 11: Studies excluded during full-text screening**

**Full text not available: n = 7**

Optimizing diagnosis of recurrent events using continuous monitoring ancillary study of Costs and effects of strategies to prevent oversedation in Intensive Care patients. Patient safety/medication safety: The impact of computerized physician order entryon medication error prevention in hospitalized patients. Health Technology Assessment Database 2005;(2). [PMID: http://onlinelibrary.wiley.com/o/cochrane/clhta/articles/HTA-32005001103/frame.html]

Patient safety/medication safety: the impact of computerized physician order entry on medication error prevention in hospitalized patients. Health Technology Assessment Database 2005;(2). [PMID: http://onlinelibrary.wiley.com/o/cochrane/clhta/articles/HTA-32005001418/frame.html]

Benoit E, Beney J. Can new technologies reduce the rate of medications errors in adult intensive care? J Pharm Belg 2011;66(3):82-91.

Bulloch MN, Olin JL. Instruments for evaluating medication use and prescribing in older adults. J Am Pharm Assoc (2003) 2014;54(5):530-7.

Salas Rojas SG, Perez Morales ME, Melendez Lopez SG, Castro Pastrana LI. Adverse drug reactions related to admissions and hospital stays: A systematic review from 2000-2011. Rev Mex Ciencia Farm 2012;43(3):19-35. [PMID: http://www.afmac.org.mx/revistas/2012/RMCF%20V43-3/ARTICULOS%20PDF]

Tipton DJ, Giannetti VJ, Kristofik JM. Managing the aftermath of medication errors: managed care's role. J Am Pharm Assoc (2003) 2003;43(5):622-9.

Vazquez CM, MartiÂ­nez Q, AA. Efficacy of medication reconciliation in the prevention of adverse events (Provisional abstract). Metas de Enfermeria 2011;14:28-32.

**Wrong setting: n = 43**

Drug groups that cause preventable hospitalisation. Aust J Pharm 2008;89(1056):77

CPOE systems can substantially reduce medication errors. Health devices 2013;42(5):169

Antia SX, Sholevar EH, Baron DA. Overdoses and ingestions of second-generation antipsychotics in children and adolescents. J Child Adolesc Psychopharmacol 2005;15(6):970-85.

Bajwa SJS, Kaur J. Clinical profile of levobupivacaine in regional anesthesia: A systematic review. J Anaesthesiol -Clin Pharmacol 2013;29(4):530-9.

Becker ML, Kallewaard M, Caspers PWJ, Schalekamp T, Stricker BHC. Potential determinants of drug-drug interaction associated dispensing in community pharmacies. Drug Saf 2005;28(5):371-8.

Beijer HJM, de Blaey CJ. Hospitalisations caused by adverse drug reactions (ADR): a meta-analysis of observational studies. Pharm World Sci 2002;24(2):46-54.

Bigham BL, Buick JE, Brooks SC, Morrison M, Shojania KG, Morrison LJ. Patient safety in emergency medical services: a systematic review of the literature. Prehosp Emerg Care 2012;16(1):20-35.

Cao LY, Taylor JS, Vidimos A. Patient safety in dermatology: A review of the literature. Dermatol Online J 2010;16(1):no. [PMID: http://dermatology.cdlib.org/1601/reviews/patient_safety/taylor.html]

Coiera E, Westbrook J, Wyatt J. The safety and quality of decision support systems. Yearb med inform 2006;20-5.

Corsonello A, Onder G, Abbatecola AM, Guffanti EE, Gareri P, Lattanzio F. Explicit criteria for potentially inappropriate medications to reduce the risk of adverse drug reactions in elderly people: From beers to STOPP/START criteria. Drug Saf 2012;35(SUPPL. 1):21-8.

Costa H, Joaquim JJ, Pires T, Matos C. Economic impact of adverse drug reactions leading to hospital admission-a systematic review. Drug Saf 2013;36(9):930

Cullinan S, O'Mahony D, Fleming A, Byrne S. A meta-synthesis of potentially inappropriate prescribing in older patients. Drugs Aging 2014;31(8):631-8.

Curtiss FR, Fry RN, Avey SG. Framework for pharmacy services quality improvement--a bridge to cross the quality chasm. Part I. The opportunity and the tool. J Manage Care Pharm 2004;10(1):60-78.

Dechanont S, Maphanta S, Butthum B, Kongkaew C. Hospital admissions/visits associated with drug-drug interactions: a systematic review and meta-analysis. Pharmacoepidemiol Drug Saf 2014;23(5):489-97.

DeFeo K, Sykora K, Eley S, Vincent D. How does pharmacogenetic testing alter the treatment course and patient response for chronic-pain patients in comparison with the current "trial-and-error" standard of care? J Am Assoc Nurse Pract 2014;26(10):530-6.

Dore DD, Hussein M, Hoffman C, Pelletier EM, Smith DB, Seeger JD. A pooled analysis of exenatide use and risk of acute pancreatitis. Curr Med Res Opin 2013;29(12):1577-86.

Einarson TR. Drug-related hospital admissions. Ann Pharmacother 1993;27(7-8):832-40.

Ferner RE, Aronson JK. Preventability of drug-related harms - part I: a systematic review. Drug Saf 2010;33(11):985-94.

Glick TH. The neurologist and patient safety. Neurolog 2005;11(3):140-9.

Hahn T, Whitbeck E. Smart pumps and synergy. A forward-thinking conversion is helping one hospital reduce i.v. medication errors and enhance staff collaboration. Nurs Manage 2007;Suppl:27-32.

Hanlon JT, Semla TP, Schmader KE. Medication misadventures in older adults: literature from 2013. J Am Geriatr Soc 2014;62(10):1950-3.

Howard RL, Avery AJ, Slavenburg S, Royal S, Pipe G, Lucassen P, et al. Which drugs cause preventable admissions to hospital? A systematic review. Br J Clin Pharmacol 2007;63(2):136-47.

Jolivot PA, Hindlet P, Pichereau C, Fernandez C, Maury E, Guidet B, et al. A systematic review of adult admissions to ICUs related to adverse drug events. Crit Care 2014;18(6):643

Kelly WN. Potential risks and prevention, Part 1: Fatal adverse drug events. Am J Health-Syst Pharm 2001;58(14):1317-24.

Khalil H, Avery AJ, Chambers H, et al. Interventions in primary care for reducing preventable medication errors that lead to hospital admissions, mortality and emergency department visits. In:2013. 11

Kongkaew C, Noyce PR, Ashcroft DM. Hospital admissions associated with adverse drug reactions: a systematic review of prospective observational studies. Ann Pharmacother 2008;42(7):1017-25.

Lainer M, Vogele A, Wensing M, Sonnichsen A. Improving medication safety in primary care. A review and consensus procedure by the LINNEAUS collaboration on patient safety in primary care. Eur J Gen Pract 2015;21 Suppl:14-8.

Lampert A, Seiberth J, Haefeli WE, Seidling HM. A systematic review of medication administration errors with transdermal patches. Expert Opin Drug Saf 2014;13(8):1101-14.

Lorenz M, Wozel G, Schmitt J. Hypersensitivity reactions to dapsone: a systematic review. Acta Derm Venereol 2012;92(2):194-9.

Madan I, Cullinan P, Ahmed SM. Occupational management of type I latex allergy. Occup Med (Oxf) 2013;63(6):395-404.

Muehlberger N, Schneeweiss S, Hasford J. Adverse drug reaction monitoring cost and benefit considerations part I: Frequency of adverse drug reactions causing hospital admissions. Pharmacoepidemiol Drug Saf 1997;6(SUPPL. 3):S71-S77

Nieuwstraten C, Labiris NR, Holbrook A. Systematic overview of drug interactions with antidepressant medications (Provisional abstract). Can J Psychiatry 2006;51:300-16.

Patel P, Zed PJ. Drug-related visits to the emergency department: how big is the problem? Pharmacotherapy 2002;22(7):915-23.

Roughead EE, Gilbert AL, Primrose JG, Sansom LN. Drug-related hospital admissions: a review of Australian studies published 1988-1996. Med J Aust 1998;168(8):405-8.

Runciman WB, Roughead EE, Semple SJ, Adams RJ. Adverse drug events and medication errors in Australia. Int J Qual Health Care 2003;15 Suppl 1:i49-i59

Saedder EA, Lisby M, Nielsen LP, Bonnerup DK, Brock B. Number of drugs most frequently found to be independent risk factors for serious adverse reactions: a systematic literature review. Br J Clin Pharmacol 2015;80(4):808-17.

Sears K, Ross-White A, Godfrey CM. The incidence, prevalence and contributing factors associated with the occurrence of medication errors for children and adults in the community setting: A systematic review. JBI Database System Rev Implement Rep 2012;10(35):2350-464. [PMID: http://www.joannabriggslibrary.org/index.php/jbisrir/article/view/35/105]

Souza TT, Godoy RR, Rotta I, Ziegelmann PK, Fernandez-Llimos F, Correr CJ. Prevalence of medication-related hospitalizations: A systematic review and meta-analysis. Int J Clin Pharm 2013;35(6):1335

Spencer R, Bell B, Avery AJ, Gookey G, Campbell SM, Royal College of General Practitioners. Identification of an updated set of prescribing--safety indicators for GPs. Br J Gen Pract 2014;64(621):e181-e190

Tache SV, Sonnichsen A, Ashcroft DM. Prevalence of adverse drug events in ambulatory care: a systematic review. Ann Pharmacother 2011;45(7-8):977-89.

Wilbur K, Hazi H, El-Bedawi A. Drug-Related Hospital Visits and Admissions Associated with Laboratory or Physiologic Abnormalities-A Systematic-Review. PloS one 2013;8(6):e66803

Winterstein AG, Sauer BC, Hepler CD, Poole C. Preventable drug-related hospital admissions. Ann Pharmacother 2002;36(7-8):1238-48.

Zed PJ, Haughn C, Black KJL, Fitzpatrick EA, Ackroyd-Stolarz S, Murphy NG, et al. Medication-related emergency department visits and hospital admissions in pediatric patients: a qualitative systematic review. J Pediatr 2013;163(2):477-83.

**Not exclusive to drug-related AEs: n = 4**

Alqubaisi M, Tonna A, Strath A, Stewart D. A systematic review of health professionals' beliefs, attitudes and experiences of medication error reporting. Int J Clin Pharm 2015;37(1):179-80.

de Vries EN, Ramrattan MA, Smorenburg SM, Gouma DJ, Boermeester MA. The incidence and nature of in-hospital adverse events: a systematic review. Qual Saf Health Care 2008 Jun;17(3):216-23. [PMID: 18519629]

Harris DD, Detke LA. The role of flooring as a design element affecting patient and healthcare worker safety. HERD 2013;6(3):95-119.

Qubaisi MA, Stewart D, Tonna A, Alison SS. Health Professionals' Beliefs, Attitudes and Experiences of Medication error reporting: A systematic review protocol. JBI Database System Rev Implement Rep 2014;12(10):109-20.

**No PADR incidence data reported: n = 129**

Paediatric pharmacovigilance. Prescrire Int 2002;11(62):183

Dosing errors appear to be the most common type of medication error in paediatric patients. Drugs Ther Perspect 2005;21(9):24-6. [PMID: http://saturn.bids.ac.uk/cgi-bin/ds_deliver/1/u/d/ISIS/21334473.1/adis/dtp/2005/00000021/00000009/art00008/930755F5E2AA55B711266579098E492EE4859ED18A.pdf?link=http://www.ingentaconnect.com/error/delivery&format=pdf]

Adverse effects: children too. Prescrire Int 2006;15(83):108

Agbabiaka TB, Savovic J, Ernst E. Methods for causality assessment of adverse drug reactions: a systematic review. Drug Saf 2008;31(1):21-37.

Ahmed Z, Barber N, Jani Y, Garfield S, Franklin BD. Economic impact of electronic prescribing in the hospital setting: A systematic review. Int J Med Inf 2016;88:1-7.

Al, Hamid A., Ghaleb, M., Aljadhey, H., et al. A systematic review of qualitative research on the contributory factors leading to medicine-related problems from the perspectives of adult patients with cardiovascular diseases and diabetes mellitus. Available at: BMJ open. [PMID:http://bmjopen.bmj.com/content/4/9/e005992.full.pdf+html]

Al SS, Stewart D. Use of the Drug Burden Index to identify and reduce potentially inappropriate prescribing of anticholinergic and sedative agents in elderly patients in institutionalized care: A systematic review protocol. JBI Database System Rev Implement Rep 2014;12(4):48-59. [PMID: http://www.joannabriggslibrary.org/index.php/jbisrir/article/view/809/1938]

Alhawassi TM, Krass I, Bajorek BV, Pont LG. A systematic review of the prevalence and risk factors for adverse drug reactions in the elderly in the acute care setting. Clin Interv Aging 2014;9:2079-86.

Alomar MJ. Factors affecting the development of adverse drug reactions (Review article). Saudi Pharm J 2014;22(2):83-94.

Alshahrani F, Marriott J, Cox A. The impact of electronic prescribing systems on the incidence of prescribing errors within in-patients settings: A systematic review. Int J Pharm Pract 2015;23:26-7.

Alsulami, Z., Conroy, S., and Choonara, I. A systematic review of the effectiveness of double checking in preventing medication errors. Available at: Archives of disease in childhood. [PMID:http://adc.bmj.com/content/97/5/e2.1.full.pdf+html]

Alsulami Z, Conroy S, Choonara I. Medication errors in the Middle East countries: a systematic review of the literature. Eur J Clin Pharmacol 2013;69(4):995-1008.

Ameer A, Ghaleb M, Dhillon S. Systematic review: Epidemiology, nature and interventions of hospital medication administration errors in paediatrics. Int J Pharm Pract 2013;21:43-4.

Ammenwerth E, Schnell-Inderst P, Machan C, Siebert U. The effect of electronic prescribing on medication errors and adverse drug events: a systematic review (DARE structured abstract). J Am Med Informatics Assoc 2008;15:585-600.

Ammenwerth E, Schnell-Inderst P, Machan C, Siebert U. The effect of electronic prescribing on medication errors and adverse drug events: a systematic review. J Am Med Inform Assoc 2008;15(5):585-600.

Aronson JK, Ferner RE. Preventability of drug-related harms - part II: proposed criteria, based on frameworks that classify adverse drug reactions. Drug Saf 2010;33(11):995-1002.

Baines RJ, Langelaan M, de Bruijne MC, Wagner C. Is researching adverse events in hospital deaths a good way to describe patient safety in hospitals: a retrospective patient record review study. BMJ open 2015;5(7):e007380

Baqir W, Crehan O, Murray R, Copeland R, Campbell D. An evaluation of pharmacist prescribing in a hospital setting. Int J Pharm Pract 2013;21:56-7.

Berdot S, Gillaizeau F, Caruba T, Prognon P, Durieux P, Sabatier B. Drug administration errors in hospital inpatients: a systematic review. PloS one 2013;8(6):e68856

Berdot S, Roudot M, Schramm C, Katsahian S, Durieux P, Sabatier B. Interventions to reduce nurses' medication administration errors in inpatient settings: A systematic review and meta-analysis. Int J Nurs Stud 2016;53:342-50.

Berhe, A., Mol, P., and Taxis, K. Medication related problems in cardio-metabolic disease management in sub-saharan africa: A systematic review. Available at: Drug safety.

Biron AD, Loiselle CG, Lavoie-Tremblay M. Work interruptions and their contribution to medication administration errors: an evidence review. Worldviews Evid Based Nurs 2009;6(2):70-86.

Brady AM, Malone AM, Fleming S. A literature review of the individual and systems factors that contribute to medication errors in nursing practice. J Nurs Manag 2009;17(6):679-97.

Carling CLL, Kirkehei I, Dalsbo TK, Paulsen E. Risks to patient safety associated with implementation of electronic applications for medication management in ambulatory care--a systematic review. BMC Med Inf Decis Mak 2013;13:133

Chapuis C, Roustit M, Bal G, Schwebel C, Pansu P, David-Tchouda S, et al. Automated drug dispensing system reduces medication errors in an intensive care setting. Crit Care Med 2010 Dec;38(12):2275-81. [PMID: 20838333]

Charles K, Cannon M, Hall R, Coustasse A. Can utilizing a computerized provider order entry (CPOE) system prevent hospital medical errors and adverse drug events? Perspect health inf manag 2014;11:1b

Charpiat B, Bedouch P, Conort O, Rose FX, Juste M, Roubille R, et al. [Opportunities for medication errors and pharmacist's interventions in the context of computerized prescription order entry: a review of data published by French hospital pharmacists]. Ann Pharm Fr 2012;70(2):62-74.

Chiatti C, Bustacchini S, Furneri G, Mantovani L, Cristiani M, Misuraca C, et al. The economic burden of inappropriate drug prescribing, lack of adherence and compliance, adverse drug events in older people: a systematic review. Drug Saf 2012;35 Suppl 1:73-87.

Chrischilles EA, Fulda TR, Byrns PJ, Winckler SC, Rupp MT, Chui MA. The role of pharmacy computer systems in preventing medication errors. J Am Pharm Assoc (Wash) 2002;42(3):439-48.

Cooper JA, Cadogan CA, Patterson SM, Kerse N, Bradley MC, Ryan C, et al. Interventions to improve the appropriate use of polypharmacy in older people: a Cochrane systematic review. BMJ open 2015;5(12):e009235

Cope JU, Rosenthal GL, Weinel P, Odegaard A, Murphy DM. FDA Safety Reviews on Drugs, Biologics, and Vaccines: 2007-2013. Pediatrics 2015;136(6):1125-31.

Dearden E, Mellanby E, Cameron H, Harden J. Which non-technical skills do junior doctors require to prescribe safely? A systematic review. Br J Clin Pharmacol 2015;80(6):1303-14.

Diav-Citrin O, Ratnapalan S, Grouhi M, Roifman C, Koren G. Medication errors in paediatrics: a case report and systematic review of risk factors. Paediatr Drugs 2000;2(3):239-42.

Ehrenstein V, Sorensen HT, Bakketeig LS, Pedersen L. Medical databases in studies of drug teratogenicity: methodological issues. Clin Epidemiol 2010;2:37-43.

El-Jardali F, Akl EA, Fadlallah R, Oliver S, Saleh N, El-Bawab L, et al. Interventions to combat or prevent drug counterfeiting: a systematic review. BMJ open 2015;5(3):e006290

Elashwah M. Medication Errors in Ambulatory Paediatric Patient Setting--How Close, or Far, are we from an Error Free Process? Infect Disord Drug Targets 2014;14(3):191-204.

Ensing HT, Stuijt CCM, van den Bemt BJF, van Dooren AA, Karapinar-Carkit F, Koster ES, et al. Identifying the Optimal Role for Pharmacists in Care Transitions: A Systematic Review. J Manag Care Spec Pharm 2015;21(8):614-36.

Espinosa-Bosch M, Santos-Ramos B, Gil-Navarro MV, Santos-Rubio MD, Marin-Gil R, Villacorta-Linaza P. Prevalence of drug interactions in hospital healthcare. Int J Clin Pharm 2012;34(6):807-17.

Fleming A, Browne J, Byrne S. The effect of interventions to reduce potentially inappropriate antibiotic prescribing in long-term care facilities: a systematic review of randomised controlled trials. Drugs Aging 2013;30(6):401-8.

Fletcher, K. E., Reed, D. A., and Arora, V. M. Patient safety, resident education and resident well-being following implementation of the 2003 ACGME duty hour rules. Available at: Journal of general internal medicine.

Fraccaro P, Arguello Casteleiro M, Ainsworth J, Buchan I. Adoption of clinical decision support in multimorbidity: a systematic review. JMIR Med Inform 2015;3(1):e4

Garfield S, Reynolds M, Dermont L, Franklin BD. Measuring the severity of prescribing errors: a systematic review. Drug Saf 2013;36(12):1151-7.

Ghaleb MA, Barber N, Franklin BD, Yeung VWS, Khaki ZF, Wong ICK. Systematic review of medication errors in pediatric patients. Ann Pharmacother 2006;40(10):1766-76.

Gillaizeau F, Chan E, Trinquart L, Colombet I, Walton RT, Rege-Walther M, et al. Computerized advice on drug dosage to improve prescribing practice. Cochrane Database Syst Rev 2013;11:CD002894

Gonzales K. Medication administration errors and the pediatric population: a systematic search of the literature. J Pediatr Nurs 2010;25(6):555-65.

Graabaek T, Kjeldsen LJ. Medication reviews by clinical pharmacists at hospitals lead to improved patient outcomes: a systematic review. Basic Clin Pharmacol Toxicol 2013;112(6):359-73.

Hajjar ER, Cafiero AC, Hanlon JT. Polypharmacy in elderly patients (DARE structured abstract). American Journal of Geriatric Pharmacotherapy 2007;5:345-51.

Hakkarainen KM, Andersson Sundell K, Petzold M, Hagg S. Methods for assessing the preventability of adverse drug events: a systematic review. Drug Saf 2012;35(2):105-26.

Harrison R, Cohen AWS, Walton M. Patient safety and quality of care in developing countries in Southeast Asia: a systematic literature review. Int J Qual Health Care 2015;27(4):240-54.

Harrison R, Walton M, Manias E, Smith-Merry J, Kelly P, Iedema R, et al. The missing evidence: a systematic review of patients' experiences of adverse events in health care. Int J Qual Health Care 2015;27(6):424-42.

Hassink J, Jansen M, Helmons P. Effects of bar code-assisted medication administration (BCMA) on frequency, type and severity of medication administration errors: A review of the literature. Euro J Hosp Pharm Sci Pra 2012;19(5):489-94. [PMID: http://ejhp.bmj.com/content/19/5/489.full.pdf+html]

Hayward, R. A., Heisler, M., Adams, J., et al. Overestimating outcome rates: Statistical estimation when reliability is suboptimal. Available at: Health services research.

Heldt T, Loss SH. Drug-nutrient interactions in the intensive care unit: literature review and current recommendations. Rev bras ter intensiva 2013;25(2):162-7.

Heneka N, Shaw T, Rowett D, Phillips JL. Quantifying the burden of opioid medication errors in adult oncology and palliative care settings: A systematic review. Palliat Med 2016;30(6):520-32.

Hertzel C, Sousa VD. The use of smart pumps for preventing medication errors. J Infus Nurs 2009;32(5):257-67.

Hill-Taylor B, Sketris I, Hayden J, Byrne S, O'Sullivan D, Christie R. Application of the STOPP/START criteria: a systematic review of the prevalence of potentially inappropriate prescribing in older adults, and evidence of clinical, humanistic and economic impact. J Clin Pharm Ther 2013;38(5):360-72.

Holland R, Desborough J, Goodyer L, Hall S, Wright D, Loke YK. Does pharmacist-led medication review help to reduce hospital admissions and deaths in older people? A systematic review and meta-analysis. Br J Clin Pharmacol 2008;65(3):303-16.

Huynh, C., Jani, Y., Tomlin, S., et al. Epidemiology of medication discrepancies upon hospital admission in children - A systematic review. Available at: Archives of disease in childhood. [PMID:http://adc.bmj.com/content/97/5/e7.1.full.pdf+html]

Ioannidis JP, Lau J. Evidence on interventions to reduce medical errors: an overview and recommendations for future research. J Gen Intern Med 2001;16(5):325-34.

Jano E, Aparasu RR. Healthcare outcomes associated with beers' criteria: a systematic review. Ann Pharmacother 2007;41(3):438-47.

Jensen LS, Merry AF, Webster CS, Weller J, Larsson L. Evidence-based strategies for preventing drug administration errors during anaesthesia. Anaesthesia 2004;59(5):493-504.

Johnson A, Guirguis E, Grace Y. Preventing medication errors in transitions of care: A patient case approach. J Am Pharm Assoc (2003) 2015;55(2):e264-e266

Johnsson A, Walter S, Grandt D, Niebling W, Gundert-Remy U. Fatal adverse drug reactions: A systematic review. Br J Clin Pharmacol 2009;68:59

Jones SW. Reducing medication administration errors in nursing practice. Nurs Stand 2009;23(50):40-6.

Kaboli PJ, Hoth AB, McClimon BJ, Schnipper JL. Clinical pharmacists and inpatient medical care: a systematic review. Arch Intern Med 2006;166(9):955-64.

Kaufmann CP, Tremp R, Hersberger KE, Lampert ML. Inappropriate prescribing: a systematic overview of published assessment tools. Eur J Clin Pharmacol 2014;70(1):1-11.

Kaufmann J, Laschat M, Wappler F. Medication errors in pediatric emergencies: a systematic analysis. Dtsch Arztebl int 2012;109(38):609-16.

Kaur S, Mitchell G, Vitetta L, Roberts MS. Interventions that can reduce inappropriate prescribing in the elderly: a systematic review. Drugs Aging 2009;26(12):1013-28.

Kaushal R, Shojania KG, Bates DW. Effects of computerized physician order entry and clinical decision support systems on medication safety: a systematic review. Arch Intern Med 2003;163(12):1409-16.

Keers RN, Williams SD, Cooke J, Ashcroft DM. Causes of medication administration errors in hospitals: a systematic review of quantitative and qualitative evidence. Drug Saf 2013;36(11):1045-67.

Keers RN, Williams SD, Cooke J, Ashcroft DM. Prevalence and nature of medication administration errors in health care settings: a systematic review of direct observational evidence. Ann Pharmacother 2013;47(2):237-56.

Keers RN, Williams SD, Cooke J, Walsh T, Ashcroft DM. Impact of interventions designed to reduce medication administration errors in hospitals: a systematic review. Drug Saf 2014;37(5):317-32.

Keijsers CJPW, van Hensbergen L, Jacobs L, Brouwers JRBJ, de Wildt DJ, ten Cate OT, et al. Geriatric pharmacology and pharmacotherapy education for health professionals and students: a systematic review. Br J Clin Pharmacol 2012;74(5):762-73.

Koumpagioti D, Varounis C, Kletsiou E, Nteli C, Matziou V. Evaluation of the medication process in pediatric patients: a meta-analysis. J Pediatr (Rio J) 2014;90(4):344-55.

Kullberg A, Larsen J, Sharp L. 'Why is there another person's name on my infusion bag?' Patient safety in chemotherapy care ? a review of the literature (Provisional abstract). European Journal of Oncology Nursing 2013;17:228-35.

Lazarou J, Pomeranz BH, Corey PN. Incidence of adverse drug reactions in hospitalized patients: a meta-analysis of prospective studies. JAMA 1998;279(15):1200-5.

Lewis PJ, Dornan T, Taylor D, Tully MP, Wass V, Ashcroft DM. Prevalence, incidence and nature of prescribing errors in hospital inpatients: a systematic review. Drug Saf 2009;32(5):379-89.

Lindsay J, Dooley M, Martin J, Fay M, Kearney A, Barras M. Reducing potentially inappropriate medications in palliative cancer patients: evidence to support deprescribing approaches. Support Care Cancer 2014;22(4):1113-9.

Liu M, McPeek Hinz ER, Matheny ME, Denny JC, Schildcrout JS, Miller RA, et al. Comparative analysis of pharmacovigilance methods in the detection of adverse drug reactions using electronic medical records. J Am Med Inform Assoc 2013;20(3):420-6.

Lucas AJ. Improving medication safety in a neonatal intensive care unit. Am J Health-Syst Pharm 2004;61(1):33-7.

Maidment ID, Haw C, Stubbs J, Fox C, Katona C, Franklin BD. Medication errors in older people with mental health problems: a review. Int J Geriatr Psychiatry 2008;23(6):564-73.

Maidment ID, Lelliott P, Paton C. Medication errors in mental healthcare: a systematic review. Qual Saf Health Care 2006;15(6):409-13.

Manley HJ, Cannella CA, Bailie GR, St Peter WL. Medication-related problems in ambulatory hemodialysis patients: a pooled analysis. Am J Kidney Dis 2005;46(4):669-80.

Mansour M, James V, Edgley A. Investigating the safety of medication administration in adult critical care settings. Nurs Crit Care 2012;17(4):189-97.

Mansouri, A., Ahmadvand, A., Hadjibabaie, M., et al. A review of medication errors in iran: Sources, underreporting reasons and preventive measures. Available at: Iranian Journal of Pharmaceutical Research. [PMID:http://ijpr.sbmu.ac.ir/?_action=showPDF&article=1431&_ob=da2217cf94675c39ea7ae5502d762f26&fileName=full_text.pdf]

Mansouri A, Ahmadvand A, Hadjibabaie M, Kargar M, Javadi M, Gholami K. Types and severity of medication errors in Iran; A review of the current literature. DARU J Pharm Sci 2013;21(1):no

Marasinghe KM. Computerised clinical decision support systems to improve medication safety in long-term care homes: a systematic review. BMJ open 2015;5(5):e006539

Marquet K, Liesenborgs A, Bergs J, Vleugels A, Claes N. Incidence and outcome of inappropriate in-hospital empiric antibiotics for severe infection: a systematic review and meta-analysis. Crit Care 2015;19:63

Martins ACM, Giordani F, Rozenfeld S. Adverse drug events among adult inpatients: a meta-analysis of observational studies. J Clin Pharm Ther 2014;39(6):609-20.

McDowell SE, Mt-Isa S, Ashby D, Ferner RE. Where errors occur in the preparation and administration of intravenous medicines: a systematic review and Bayesian analysis. Qual Saf Health Care 2010;19(4):341-5.

McLeod MC, Barber N, Franklin BD. Methodological variations and their effects on reported medication administration error rates. BMJ Qual Saf 2013;22(4):278-89.

Mekonnen AB, McLachlan AJ, Brien JA. Pharmacy-led medication reconciliation programmes at hospital transitions: a systematic review and meta-analysis. J Clin Pharm Ther 2016;41(2):128-44.

Metsala E, Vaherkoski U. Medication errors in elderly acute care--a systematic review. Scand J Caring Sci 2014;28(1):12-28.

Miguel A, Azevedo LF, Araujo M, Pereira AC. Frequency of adverse drug reactions in hospitalized patients: a systematic review and meta-analysis. Pharmacoepidemiol Drug Saf 2012;21(11):1139-54.

Mueller SK, Sponsler KC, Kripalani S, Schnipper JL. Hospital-based medication reconciliation practices: a systematic review (Provisional abstract). Arch Intern Med 2012;172:e1-e14

Mueller SK, Sponsler KC, Kripalani S, Schnipper JL. Hospital-based medication reconciliation practices: a systematic review. Arch Intern Med 2012;172(14):1057-69.

Patel S, Loveridge R. Obstetric neuraxial drug administration errors: A quantitative and qualitative analytical review. Anesth Analg 2015;121(6):1570-7. [PMID: http://journals.lww.com/anesthesia-analgesia/toc/publishahead]

Patel TK, Patel PB. Incidence of Adverse Drug Reactions in Indian Hospitals: A Systematic Review of Prospective Studies. Curr Drug Saf 2016;11(2):128-36.

Porterfield A, Engelbert K, Coustasse A. Electronic prescribing: improving the efficiency and accuracy of prescribing in the ambulatory care setting. Perspect health inf manag 2014;11:1g

Raban MZ, Westbrook JI. Are interventions to reduce interruptions and errors during medication administration effective?: a systematic review. BMJ Qual Saf 2014;23(5):414-21.

Radley DC, Wasserman MR, Olsho LE, Shoemaker SJ, Spranca MD, Bradshaw B. Reduction in medication errors in hospitals due to adoption of computerized provider order entry systems. J Am Med Inform Assoc 2013;20(3):470-6.

Reckmann MH, Westbrook JI, Koh Y, Lo C, Day RO. Does computerized provider order entry reduce prescribing errors for hospital inpatients? A systematic review. J Am Med Inform Assoc 2009;16(5):613-23.

Ross S, Bond C, Rothnie H, Thomas S, Macleod MJ. What is the scale of prescribing errors committed by junior doctors? A systematic review. Br J Clin Pharmacol 2009;67(6):629-40.

Rosse F, Maat B, Rademaker CM, Vught AJ, Egberts AC, Bollen CW. The effect of computerized physician order entry on medication prescription errors and clinical outcome in pediatric and intensive care: a systematic review (DARE structured abstract). Pediatrics 2009;123:1184-90.

Santos AP, da Silva DT, dos Santos Junior GA, Silvestre CC, Nunes MAP, Lyra DPJ, et al. Evaluation of the heterogeneity of studies estimating the association between risk factors and the use of potentially inappropriate drug therapy for the elderly: a systematic review with meta-analysis. Eur J Clin Pharmacol 2015;71(9):1037-50.

Schedlbauer A, Prasad V, Mulvaney C, Phansalkar S, Stanton W, Bates DW, et al. What evidence supports the use of computerized alerts and prompts to improve clinicians' prescribing behavior? J Am Med Inform Assoc 2009;16(4):531-8.

Shimura CN, Da SJ. Microbial resistance or team resistance? BMC Proc 2011;5:no

Shojania KG, Duncan BW, McDonald KM, Wachter RM, Markowitz AJ. Making health care safer: a critical analysis of patient safety practices. Evid Rep Technol Assess (Summ ) 2001;(43):i-668. [PMID: 11510252]

Sinnemaki J, Sihvo S, Isojarvi J, Blom M, Airaksinen M, Mantyla A. Automated dose dispensing service for primary healthcare patients: a systematic review. Syst rev 2013;2:1

Smeulers M, Verweij L, Maaskant JM, De BM, Krediet CTP, Nieveen Van Dijkum EJM, et al. Quality Indicators for safe medication preparation and administration: A systematic review. PloS one 2015;10(4):no. [PMID: http://www.plosone.org/article/fetchObject.action?uri=info:doi/10.1371/journal.pone.0122695&representation=PDF]

Snijders C, van Lingen RA, Molendijk A, Fetter WPF. Incidents and errors in neonatal intensive care: a review of the literature. Arch Dis Child Fetal Neonatal Ed 2007;92(5):F391-F398

Stang AS, Wingert AS, Hartling L, Plint AC. Adverse events related to emergency department care: a systematic review. PloS one 2013;8(9):e74214

Sturzlinger H, Hiebinger C, Pertl D, Traurig P. Computerized Physician Order Entry - effectiveness and efficiency of electronic medication ordering with decision support systems. GMS Health Technol Assess 2009;5:Doc07

Tam VC, Knowles SR, Cornish PL, Fine N, Marchesano R, Etchells EE. Frequency, type and clinical importance of medication history errors at admission to hospital: a systematic review. CMAJ 2005;173(5):510-5.

Thomas RE. Assessing Medication Problems in those > 65 Using the STOPP and START Criteria. Curr Aging Sci 2016;9(2):150-8.

Tuccori M, Convertino I, Capogrosso SA, Mantarro S, Marino A, Montagnani S, et al. Estimation of theoretical cost preventability achievable with an effective pharmacovigilance activity in a pharmacovigilance regional centre in Italy. Drug Saf 2015;38(10):1033-4.

Tully MP, Seston EM. Impact of pharmacists providing a prescription review and monitoring service in ambulatory care or community practice. Ann Pharmacother 2000;34(11):1320-31.

Tully MP, Ashcroft DM, Dornan T, Lewis PJ, Taylor D, Wass V. The causes of and factors associated with prescribing errors in hospital inpatients: a systematic review. Drug Saf 2009;32(10):819-36.

Valuck, R. J., Byrns, P. J., Fulda, T. R., et al. Methodology for assessing drug-drug interaction evidence in the peer-reviewed medical literature. Available at: Current Therapeutic Research - Clinical and Experimental.

van Rosse F, Maat B, Rademaker CMA, van Vught AJ, Egberts ACG, Bollen CW. The effect of computerized physician order entry on medication prescription errors and clinical outcome in pediatric and intensive care: a systematic review. Pediatrics 2009;123(4):1184-90.

Vlayen A, Verelst S, Bekkering GE, Schrooten W, Hellings J, Claes N. Incidence and preventability of adverse events requiring intensive care admission: a systematic review. J Eval Clin Pract 2012 Apr;18(2):485-97. [PMID: 21210898]

Wiffen P, Gill M, Edwards J, Moore A. Adverse drug reactions in hospital patients: a systematic review of the prospective and retrospective studies (Provisional abstract). Bandolier 2002;June:1-14.

Wilson S, Bremner A, Hauck Y, Finn J. The effect of nurse staffing on clinical outcomes of children in hospital: a systematic review. Int j evid -based healthc 2011;9(2):97-121.

Wong K, Yu SK, Holbrook A. A systematic review of medication safety outcomes related to drug interaction software (DARE structured abstract). J Popul ther Clin Pharmacol 2010;17:e243-e255

Wong K, Yu SKH, Holbrook A. A systematic review of medication safety outcomes related to drug interaction software. J Popul Ther Clin Pharmacol 2010;17(2):e243-e255

Wulff K, Cummings GG, Marck P, Yurtseven O. Medication administration technologies and patient safety: a mixed-method systematic review. J Adv Nurs 2011;67(10):2080-95.

Yang C, Yang L, Xiang X, Tang Y, Wang H, Bobai N, et al. Interventions Assessment of Prescription Automatic Screening System in Chinese Hospitals: A Systematic Review. Drug Inf J 2012;46(6):669-76.

Yip VL, Marson AG, Jorgensen AL, Pirmohamed M, Alfirevic A. HLA genotype and carbamazepine-induced cutaneous adverse drug reactions: a systematic review (Provisional abstract). Clin Pharmacol Ther 2012;92:757-65.

Young J, Slebodnik M, Sands L. Bar code technology and medication administration error. J Patient Saf 2010;6(2):115-20.

**Not a systematic review: n = 49**

Alanazi MA, Tully MP, Lewis PJ. A systematic review of the prevalence and incidence of prescribing errors with high-risk medicines in hospitals. J Clin Pharm Ther 2016;41(3):239-45.

Cano FG, Rozenfeld S. Adverse drug events in hospitals: a systematic review. Cad Saude Publica 2009;25 Suppl 3:S360-S372

Clyne B, Bradley MC, Hughes C, Fahey T, Lapane KL. Electronic prescribing and other forms of technology to reduce inappropriate medication use and polypharmacy in older people: a review of current evidence. Clin Geriatr Med 2012;28(2):301-22.

Di Giorgio C, Provenzani A, Polidori P. Potentially inappropriate drug prescribing in elderly hospitalized patients: an analysis and comparison of explicit criteria. Int J Clin Pharm 2016;38(2):462-8.

Faria R, Barbieri M, Light K, Elliott RA, Sculpher M. The economics of medicines optimization: policy developments, remaining challenges and research priorities. Br Med Bull 2014;111(1):45-61.

Fialova, D. Medication errors in elderly population. Available at: Basic and Clinical Pharmacology and Toxicology.

Franklin B, Reynolds M. A comparative study of prescribing errors in three NHS organisations. Int J Pharm Pract 2010;18:80-1.

Frith KH. Medication errors in the intensive care unit: literature review using the SEIPS model. AACN Adv Crit Care 2013;24(4):389-404.

Galadanci, H. S. Protecting patient safety in resource-poor settings. Available at: Best Practice and Research: Clinical Obstetrics and Gynaecology.

Georgiou A, Prgomet M, Paoloni R, Creswick N, Hordern A, Walter S, et al. The effect of computerized provider order entry systems on clinical care and work processes in emergency departments: a systematic review of the quantitative literature. Ann Emerg Med 2013;61(6):644-53.

Goettler, M., Schneeweiss, S., and Hasford, J. Adverse drug reaction monitoring - Cost and benefit considerations part II: Cost and preventability of adverse drug reactions leading to hospital admission. Available at: Pharmacoepidemiology and drug safety.

Heneka N, Phillips JL, Rowett D, Shaw T. Identifying opioid medication error types, incidence and patient impact in adult oncology and palliative care settings: A systematic review. Asia-Pac J Clin Oncol 2015;11:128

Impicciatore P, Choonara I, Clarkson A, Provasi D, Pandolfini C, Bonati M. Incidence of adverse drug reactions in paediatric in/out-patients: a systematic review and meta-analysis of prospective studies. Br J Clin Pharmacol 2001;52(1):77-83.

Impicciatore P, Choonara I, Clarkson A, Provasi D, Pandolfini C, Bonati M. Incidence of adverse drug reactions in paediatric in/out-patients: a systematic review and meta-analysis of prospective studies. Br J Clin Pharmacol 2001 Jul;52(1):77-83. [PMID: 11453893]

Kanjanarat P, Winterstein AG, Johns TE, Hatton RC, Gonzalez-Rothi R, Segal R. Nature of preventable adverse drug events in hospitals: a literature review. Am J Health Syst Pharm 2003 Sep 1;60(17):1750-9. [PMID: 14503111]

Karnon J, McIntosh A, Dean J, Bath P, Hutchinson A, Oakley J, et al. Modelling the expected net benefits of interventions to reduce the burden of medication errors. J Health Serv Res Policy 2008;13(2):85-91.

Kazandjian VA, Matthes N, Thomas T. Errors: can indicators measure the magnitude? J Eval Clin Pract 2001;7(2):253-60.

Khan LM. Comparative epidemiology of hospital-acquired adverse drug reactions in adults and children and their impact on cost and hospital stay--a systematic review. Eur J Clin Pharmacol 2013;69(12):1985-96.

Kiekkas P, Karga M, Lemonidou C, Aretha D, Karanikolas M. Medication errors in critically ill adults: a review of direct observation evidence. Am J Crit Care 2011;20(1):36-44.

Krahenbuhl-Melcher A, Schlienger R, Lampert M, Haschke M, Drewe J, Krahenbuhl S. Drug-related problems in hospitals: a review of the recent literature. Drug Saf 2007;30(5):379-407.

Kremsdorf R. Using innovation technology to enhance patient care delivery. Nurs Outlook 2003;51(3):S16-S20

Lehnbom EC, Stewart MJ, Manias E, Westbrook JI. The impact of medication reconciliation and review on clinical outcomes (Provisional abstract). Database of Abstracts of Reviews of Effects 2014;1298-312.

Lehnbom EC, Stewart MJ, Manias E, Westbrook JI. Impact of medication reconciliation and review on clinical outcomes. Ann Pharmacother 2014;48(10):1298-312.

Lindell-Osuagwu L, Korhonen MJ, Saano S, Helin-Tanninen M, Naaranlahti T, Kokki H. Off-label and unlicensed drug prescribing in three paediatric wards in Finland and review of the international literature. J Clin Pharm Ther 2009 Jun;34(3):277-87. [PMID: PM:19650250]

Lisby M, Nielsen LP, Brock B, Mainz J. Focused Conference Group: P13 - Maximising benefits and minimizing harms from drugs does definition of medication errors have any impact at prevalence? A systematic review of definitions. Basic Clin Pharmacol Toxicol 2010;107:417

MacFie, C. C., Baudouin, S. V., and Messer, P. B. An integrative review of drug errors in critical care. Available at: Journal of the Intensive Care Society. [PMID:http://inc.sagepub.com/content/17/1/63.full.pdf]

Miller MR, Robinson KA, Lubomski LH, Rinke ML, Pronovost PJ. Medication errors in paediatric care: a systematic review of epidemiology and an evaluation of evidence supporting reduction strategy recommendations. Qual Saf Health Care 2007;16(2):116-26.

Nivya K, Sri SK, V, Ragoo N, Jayaprakash B, Sonal SM. Systemic review on drug related hospital admissions - A pubmed based search. Saudi Pharm J 2015;23(1):1-8. [PMID: http://www.sciencedirect.com/science/journal/13190164]

Nor Aripin, K. N. B., Sammons, H. M., and Choonara, I. Focused Conference Group: P10 - Drugs for half the world: Paediatric clinical pharmacology safety in paediatric drug trials - An update from randomised controlled trials published in 2007. Available at: Basic and Clinical Pharmacology and Toxicology.

O'Malley P. Think bar-code medication administration eliminates adverse drug events? Think again! Clin Nurse Spec 2008;22(6):269-70.

Ohashi K, Dalleur O, Dykes PC, Bates DW. Benefits and risks of using smart pumps to reduce medication error rates: a systematic review. Drug Saf 2014;37(12):1011-20.

Oren E, Shaffer ER, Guglielmo BJ. Impact of emerging technologies on medication errors and adverse drug events. Am J Health-Syst Pharm 2003;60(14):1447-58.

Paran Y, Mashav N, Henis O, Swartzon M, Arbel Y, Justo D. Drug-induced torsades de pointes in patients aged 80 years or more. Anadolu Kardiyol Derg 2008;8(4):260-5.

Poudel A, Ballokova A, Hubbard RE, Gray LC, Mitchell CA, Nissen LM, et al. Algorithm of medication review in frail older people: Focus on minimizing the use of high-risk medications. Geriatr Gerontol Int 2015;

Procyshyn RM, Barr AM, Brickell T, Honer WG. Medication errors in psychiatry: a comprehensive review. CNS drugs 2010;24(7):595-609.

Qureshi NA, Neyaz Y, Khoja T, Magzoub MA, Haycox A, Walley T. Physicians' medication prescribing in primary care in Riyadh city, Saudi Arabia. Literature review, part 3: Prescribing errors. East Mediterr Health J 2011;17(2):140-8. [PMID: http://www.emro.who.int/emhj/V17/02/17_2_2011_0140_0148.pdf]

Saedder EA, Brock B, Nielsen LP, Bonnerup DK, Lisby M. Identifying high-risk medication: a systematic literature review. Eur J Clin Pharmacol 2014;70(6):637-45.

Sanghera N, Chan PY, Khaki ZF, Planner C, Lee KKC, Cranswick NE, et al. Interventions of hospital pharmacists in improving drug therapy in children: a systematic literature review. Drug Saf 2006;29(11):1031-47.

Shamliyan TA, Duval S, Du J, Kane RL. Just what the doctor ordered. Review of the evidence of the impact of computerized physician order entry system on medication errors. Health Serv Res 2008;43(1 Pt 1):32-53.

Shuster J. Controversial ADR meta-analysis; severe respiratory distress associated with amphotericin B lipid complex; mood disorder caused by furazolidone; liver failure related to pemoline; toxicity from OTC chromium; azathioprine hypersensitivity. Hosp Pharm 1998;33(7):826-7.

Sultana J, Spina E, Trifiro G. Antidepressant use in the elderly: the role of pharmacodynamics and pharmacokinetics in drug safety. Expert Opin Drug Metab Toxicol 2015;11(6):883-92.

Thomsen LA, Winterstein AG, Sondergaard B, Haugbolle LS, Melander A. Systematic review of the incidence and characteristics of preventable adverse drug events in ambulatory care. Ann Pharmacother 2007;41(9):1411-26.

van den Bemt PMLA, Idzinga JC, Robertz H, Kormelink DG, Pels N. Medication Administration Errors in Nursing Homes Using an Automated Medication Dispensing System. J Am Med Informatics Assoc 2009;16(4):486-92.

von Laue NC, Schwappach DLB, Koeck CM. The epidemiology of preventable adverse drug events: a review of the literature. Wien Klin Wochenschr 2003;115(12):407-15.

Voshall B, Piscotty R, Lawrence J, Targosz M. Barcode medication administration work-arounds: a systematic review and implications for nurse executives. J Nurs Adm 2013;43(10):530-5.

Wilmer A, Louie K, Dodek P, Wong H, Ayas N. Incidence of medication errors and adverse drug events in the ICU: a systematic review. Qual Saf Health Care 2010;19(5):e7

Wiltink EH. Anticoagulant therapy: We have to do better! A systematic review. Euro J Hosp Pharm Sci Pra 2016;21(2):108-12. [PMID: http://ejhp.bmj.com/content/by/year/2015]

Wright K. Do calculation errors by nurses cause medication errors in clinical practice? A literature review. Nurse Educ Today 2010;30(1):85-97.

Zernikow B, Michel E, Fleischhack G, Bode U. Accidental iatrogenic intoxications by cytotoxic drugs: error analysis and practical preventive strategies. Drug Saf 1999;21(1):57-74.
